# Supplementary material for: Continental-scale drivers of soil microbial extracellular polymeric substances
Source: Nat Commun. 2026 Mar 2;17:3334. doi: 10.1038/s41467-026-70068-0 (PMC13066494; doi:10.1038/s41467-026-70068-0)
Supplement: Supplementary file 1 — Supplementary Information [file 41467_2026_70068_MOESM1_ESM.pdf]

Supplementary Information for

Continental-scale drivers of soil microbial extracellular polymeric substances

Ke Shi *et al.*

*Corresponding author:

Honghua Ruan. Email: hhruan@njfu.edu.cn
Wolfgang Wanek. Email: wolfgang.wanek@univie.ac.at

This PDF file includes:

Supplementary Method 1
Supplementary Discussion 1 to 3
Supplementary Figures 1 to 7
Supplementary Tables 1 to 19
Supplementary References

CONTENTS

Supplementary Method 1: Optimizing EPS extraction across bedrock and SOC gradients 5

Supplementary Discussion 1: Mechanisms of EPS - soil particle associations.................................6

Supplementary Discussion 2: Land use effects on soil EPS contents and EPS-C/MBC ratios.........7

Supplementary Discussion 3: Bedrock vs. land use: drivers of specific EPS components...............8

Supplementary Figures 9

Supplementary Tables...16

Supplementary References 78

Abbreviations

EPS variables:
EPS, extracellular polymeric substances
EPS-C, extracellular polymeric substances carbon
EPS-C/SOC, extracellular polymeric substances carbon to soil organic carbon ratio
EPS-C/MBC, extracellular polymeric substances carbon to microbial biomass carbon ratio
EPS PN, extracellular polymeric substances proteins
EPS PS, extracellular polymeric substances polysaccharides
EPS PN/PS ratio, EPS proteins to EPS polysaccharides ratio

Climate factors:
MAT, mean annual temperature
MAP, mean annual precipitation
PET, potential evapotranspiration
ADI, aridity index

Plant factors:
FRB, fine root biomass
Root C, root carbon content
Root N, root nitrogen content
Root P, root phosphorus content
Root C/N, root carbon to nitrogen ratio
Root C/P, root carbon to phosphorus ratio
Root N/P, root nitrogen to phosphorus ratio

Microbial factors:
MBC, microbial biomass carbon
MBN, microbial biomass nitrogen
MBP, microbial biomass phosphorus
CUE, microbial carbon use efficiency
NUE, microbial nitrogen use efficiency
C_{growth}, microbial growth
q_{Growth}, microbial growth normalized to microbial biomass carbon
MBT, microbial biomass turnover time
VectorL, enzyme vector length
VectorA, enzyme vector angle
GPB, gram-positive bacteria PLFA biomass
GNB, gram-negative bacteria PLFA biomass
Fungi, fungal PLFA biomass
Bacteria, bacterial PLFA biomass
PLFA, phospholipid fatty acids

B/F, bacteria to fungi ratio
BG, β-glucosidase
NAG, N-acetyl-β-glucosaminidase
LAP, leucine aminopeptidase
AP, acid phosphatase
CEL, cellobiosidase
BX, β-xylosidase
POX, phenoloxidase

Soil factors:
SOC, soil organic carbon
TN, total nitrogen
TP, total phosphorus
C/N, soil carbon to nitrogen ratio
C/P, soil carbon to phosphorus ratio
N/P, soil nitrogen to phosphorus ratio
NH_4^+, ammonia nitrogen
NO_3^-, nitrate nitrogen
TDN, total dissolved nitrogen
DOC, dissolved organic carbon
TOP, total organic phosphorus
WHC, water holding capacity
CEC, cation exchange capacity
SMC, soil moisture content
Ca_e, exchangeable calcium (Ca^{2+})
Mg_e, exchangeable magnesium (Mg^{2+})
Fe_d, iron (Fe) oxyhydroxides extracted with Na-dithionite
Fe_c, crystalline iron (Fe) oxyhydroxides
Al_o, aluminum (Al) oxyhydroxides extracted with acid ammonium oxalate
Fe_o, iron (Fe) oxyhydroxides extracted with acid ammonium oxalate
Al_d, aluminum (Al) oxyhydroxides extracted with Na-dithionite

Supplementary Method 1: Optimizing EPS extraction across bedrock and SOC gradients

Before EPS quantification, a preliminary experiment was conducted to test EPS extraction efficiency with different CER:soil ratios across bedrock types, considering the expected higher exchangeable Ca^{2+} levels in carbonate soils and the wide range of SOC concentration variations due to the extensive sampling range. Specifically, three carbonate soil samples and three non-carbonate soil samples with high, medium, and low SOC contents were selected. For each sample, 0.5 g, 1 g, 2 g, and 3 g of air-dried soil were weighed, and then 10 g of CER was added for extraction, to demonstrate that the CER method and dosage used are suitable for different types of soil samples with varying SOC contents. This experiment showed that using 1 g of air-dried soil with 10 g of CER provided a good balance for determining soil EPS, as 3 g was more effective for polysaccharides and 0.5 g or 1 g for proteins, regardless of bedrock type or SOC level (Supplementary Fig. 4).

Supplementary Discussion 1: Mechanisms of EPS - soil particle associations

EPS in soils is thought to associate with soil particles through multiple mechanisms, including (i) physical occlusion inside microaggregates, (ii) co-precipitation and complexation with Fe/Al, (iii) hydrogen bonding, (iv) hydrophobic interactions, (v) inner-sphere ligand exchange to Fe/Al (oxyhydr)oxides and clays, (vi) outer-sphere electrostatic attraction, and (vii) multivalent cation bridging between negatively charged sites of EPS and those on other polymers and minerals[1, 2, 3]. The relative importance of these mechanisms is suggested to differ among major EPS compound classes, with EPS polysaccharides primarily binding via mechanisms (ii, iii, v, vii), and both can promote aggregate formation and are therefore affected (i) and can be occluded in microaggregates[1, 2, 3].

Cation bridging via Ca^{2+} (vii) and electrostatic attraction (vi) are generally considered to result in relatively loose and reversible associations. This EPS fraction is therefore assumed to be preferentially targeted by the CER extraction method, as Ca^{2+} removal and changes in ionic strength weaken these interactions. In contrast, EPS associated with soil particles via other mechanisms is thought to result in weaker associations (e.g., hydrogen bonding; iii) or stronger interactions (e.g., co-precipitation, hydrophobic interactions, and inner-sphere complexation; ii, iv, v), but these fractions are not expected to be efficiently extracted by CER[1, 2, 3].

Because comprehensive studies covering different EPS components, sources, and soil types are still lacking, the relative contribution of these EPS-soil association mechanisms remains poorly constrained.

Supplementary Discussion 2: Land use effects on soil EPS contents and EPS-C/MBC ratios

Among the three land-use types, EPS protein content differed significantly, being highest in grassland soils, whereas EPS polysaccharide and total EPS contents did not differ significantly (Figs. 2, 3). To identify potential drivers of EPS content variation, we found that MBC was significantly affected by land use and showed a pattern consistent with EPS (Supplementary Tables 4, 6, 7). This, together with the positive correlation between MBC and EPS (Fig. 2c), suggests that higher MBC is associated with higher EPS content across land-use types. In addition, land-use exerted a stronger influence on soil nutrient status (e.g., SOC, TOP, TP, Mg_e, and NO_3^-) and fine root traits (FRB and Root N) than bedrock (Supplementary Tables 4, 6). Among these, SOC and TOP were significantly higher in grassland than in cropland and positively correlated with EPS, while root N was also highest in grasslands, whereas FRB peaked in woodlands but remained relatively high in grasslands. These indicators, such as nutrient availability and plant inputs (root turnover and exudation), are linked to higher EPS content as observed by others[4]. This interpretation is consistent with previous findings: Redmile-Gordon et al.[5] observed the highest EPS contents in grasslands, while Kidinda et al.[6] reported higher EPS in fertilized croplands than in forests on nutrient-poor tropical soils. Together, these studies suggest that land-use effects on EPS are primarily mediated by plant inputs and nutrient availability. Conversely, the lowest MBC and SOC observed in cropland soils may reflect soil degradation or organic matter depletion caused by long-term intensive management practices such as tillage, which could, in turn, contribute to the reduced EPS levels. For instance, frequent tillage can disrupt soil structure and aggregates, thereby reducing soil organic matter content[7]. On the other hand, although croplands had the highest clay and exchangeable Ca^{2+} contents, which provide greater sorptive surface area and sorption potential for EPS, their EPS levels were lowest. This suggests that limited labile C availability, linked to lower SOC and FRB in croplands, may play a more decisive role in constraining microbial EPS content across the investigated soils. Substantial differences in soil microbial communities under different land use types[8] may also be associated with the observed EPS pool[9]. While microbial diversity was not investigated in this study, in accordance with investigations of legacy vs current management effects on EPS[5], it is primarily the interplay between plant inputs and anthropogenic management that causes these differences in total EPS and EPS protein contents[5].

Supplementary Discussion 3: Bedrock vs. land use: drivers of specific EPS components

A more detailed analysis revealed that it was primarily the bedrock type that affected the contents of EPS polysaccharides, while the type of land use influenced the contents of EPS proteins (Fig. 3). This divergence likely stems from their distinct environmental dependencies and biosynthetic pathways. Based on our data, bedrock type significantly influenced microbial biomass (MBC and MBN), soil texture (clay content), CEC, and exchangeable Ca^{2+} (Supplementary Table 4), all of which are positively associated with EPS polysaccharides content. In contrast, land use significantly influenced MBN and had a stronger effect on microbial q_{Growth} and soil nutrient availability, such as soil NH_4^+, NO_3^-, and TOP, as well as on SOC (Supplementary Table 6), which plays a critical role in microbial protein synthesis and was positively related to EPS protein. This is consistent with our random forest analysis showing that EPS polysaccharides were mainly driven by Ca_e, MBN, and SOC, whereas EPS proteins were predominantly influenced by MBN, SOC, and q_{Growth} (Fig. 4). These results indicate that while both EPS polysaccharides and EPS proteins are influenced by microbial abundance and nitrogen availability (MBN) as well as soil C status (SOC), EPS polysaccharides are closely associated with soil Ca^{2+}, and EPS proteins are more strongly linked to microbial biosynthetic activity (q_{Growth}). Additionally, based on the principle of the CER method, variations in Ca^{2+} concentrations induced by different bedrock types may influence EPS extraction efficiency and thus affect the measured EPS contents[10]. Although a preliminary experiment was conducted to optimize the extraction procedure across soil types, variability in extraction efficiency cannot be fully excluded. However, since only EPS polysaccharides, but not EPS proteins, showed significant variation with bedrock type, this indicates that the contrasting patterns are unlikely to result solely from methodological bias, but rather reflect differences in environmental or microbial regulation of EPS components.

Supplementary Figures

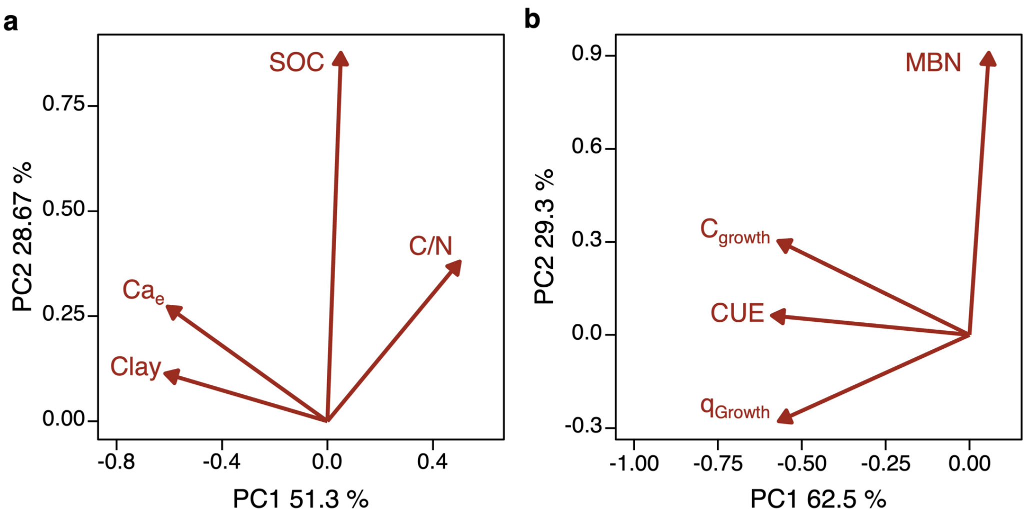

Supplementary Figure 1 | Principal component analysis (PCA) of soil and microbial composite factors. a PCA loading plot of soil variables. **b** PCA loading plot of microbial variables. Red arrows indicate PCA loading vectors. SOC, soil organic carbon; Ca_e, exchangeable calcium (Ca^{2+}); C/N, soil carbon to nitrogen ratio; MBN, microbial biomass nitrogen; CUE, microbial carbon use efficiency; C_{growth}, microbial growth; q_{Growth}, microbial growth normalized to microbial biomass carbon.

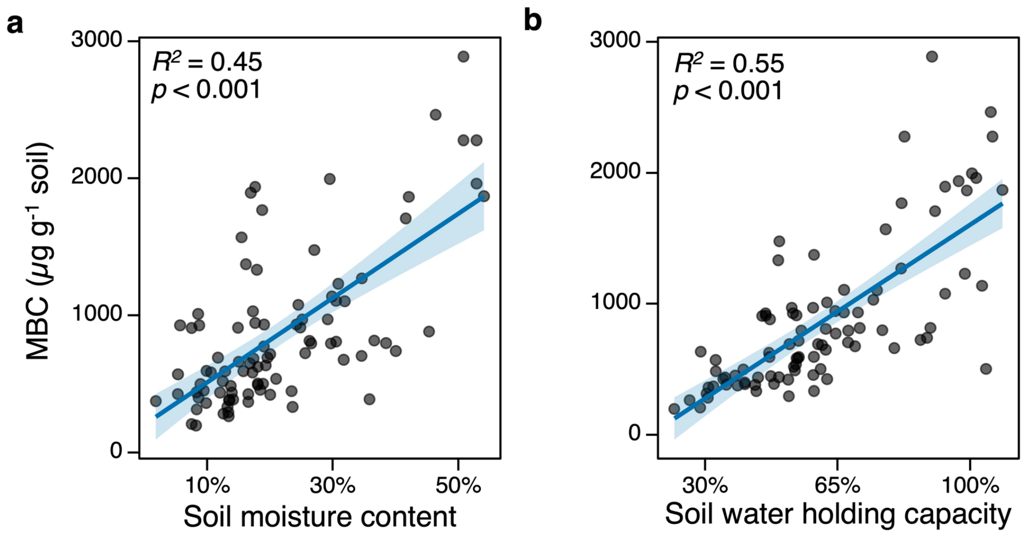

Supplementary Figure 2 | Linear regressions between microbial biomass carbon (MBC) and soil water properties. a Relationship between MBC and soil moisture content. **b** Relationship between MBC and water holding capacity. Each point represents an individual soil sample. Blue solid lines indicate linear regression fits and shaded areas represent 95% confidence intervals; R^2 and p-value are shown in each panel.

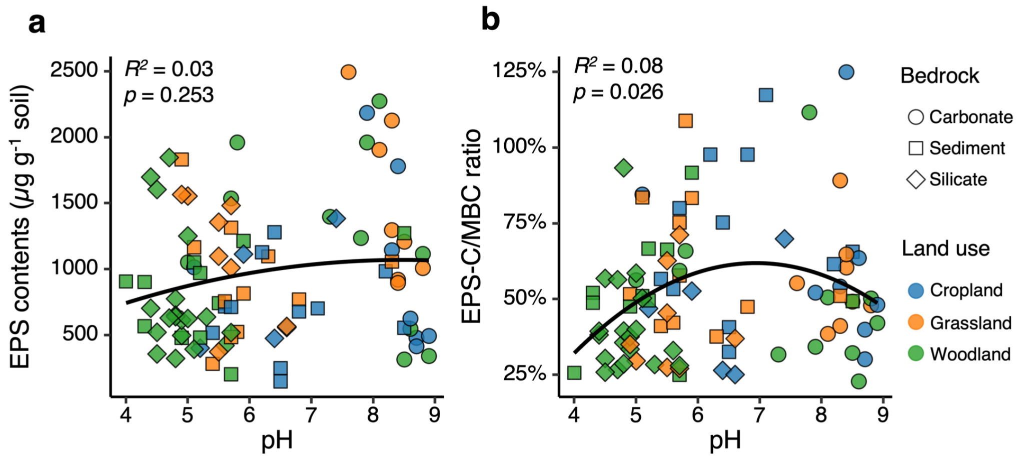

Supplementary Figure 3 | Relationship between extracellular polymeric substances (EPS) content, EPS carbon to microbial biomass carbon (EPS-C/MBC) ratio and soil pH. a Relationship between EPS and soil pH. **b** Relationship between EPS-C/MBC and soil pH. Each point represents an individual soil sample. Black lines indicate quadratic regression fits.

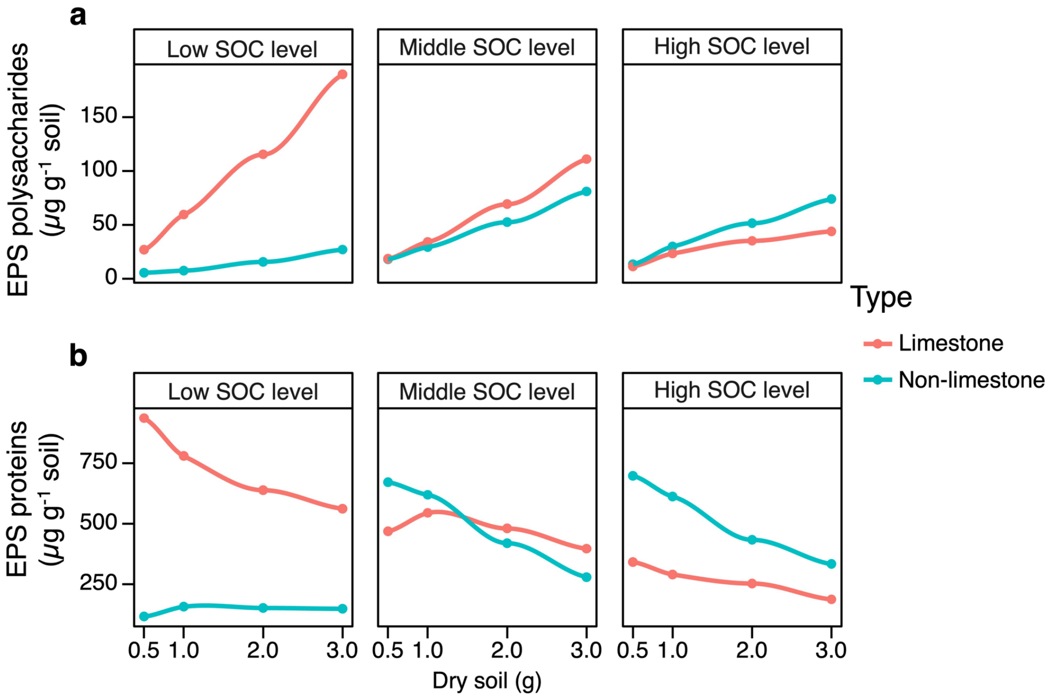

Supplementary Figure 4 | Extracellular polymeric substances (EPS) extracted from soils with different bedrock types and soil organic carbon (SOC) levels. a EPS polysaccharides measured with increasing soil mass under different SOC levels. **b** EPS proteins measured with increasing soil mass under different SOC levels.

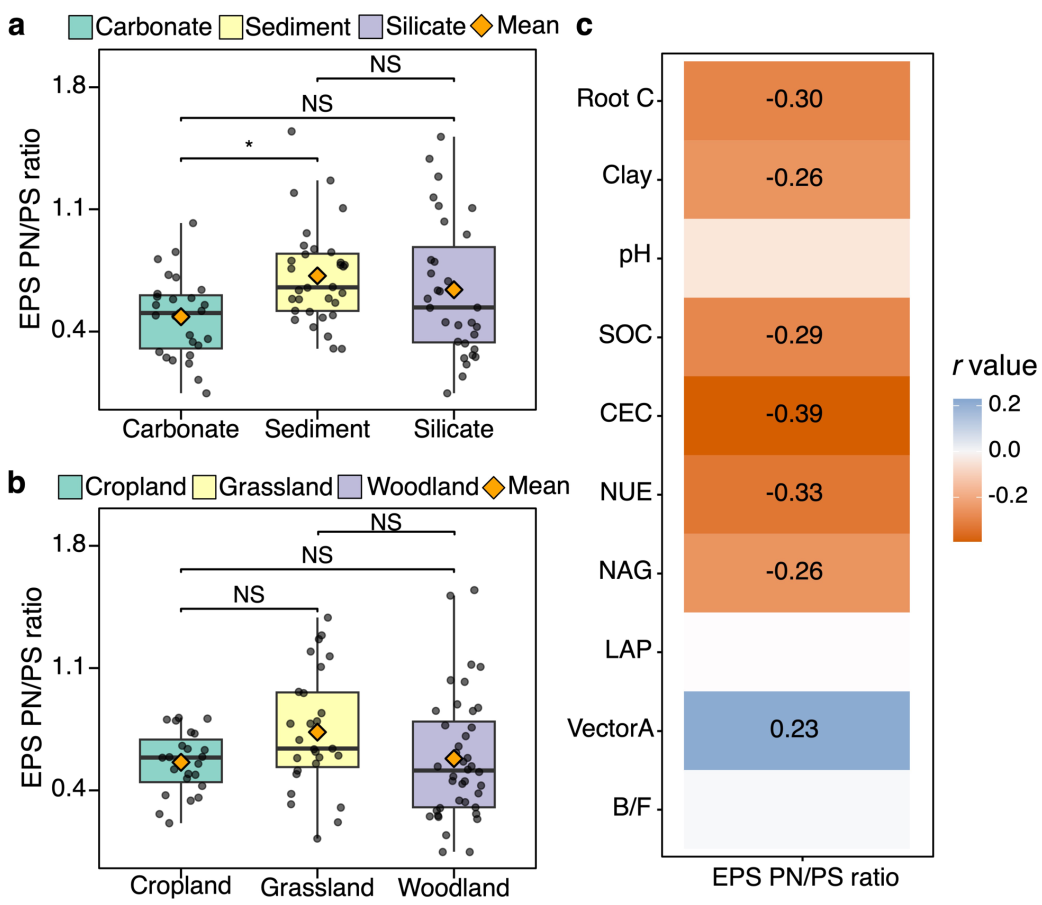

Supplementary Figure 5 | Soil extracellular polymeric substances (EPS) proteins (EPS PN) to polysaccharides (EPS PS) ratio (EPS PN/PS) and their correlations with environmental and microbial factors. a Box plots of EPS PN/PS ratio in soils from different bedrock types. **b** Box plots of EPS PN/PS ratio in soils from different land use types. Individual data points are displayed as dots with jitter to show distribution. Boxes show the median (middle line), 25th and 75th percentiles (interquartile range), and whiskers represent the 5th and 95th percentiles. Significant differences between groups are indicated on the plot. * $p < 0.05$, ** $p < 0.01$, *** $p < 0.001$, NS $p > 0.05$. **c** Heatmap showing the correlations between EPS PN/PS ratio with environmental and microbial variables. Only significant correlations ($p < 0.05$) are shown, except for pH, leucine aminopeptidase (LAP), and bacterial to fungal ratio (B/F), which are displayed regardless of significance. Numbers in the heatmap represent correlation coefficients (r); variables without numbers are non-significant ($p > 0.05$). Root C, root carbon content; SOC, soil organic carbon; CEC, cation exchange capacity; NUE, microbial nitrogen use efficiency; NAG, N-acetyl-β-glucosaminidase; VectorA, enzyme vector angle.

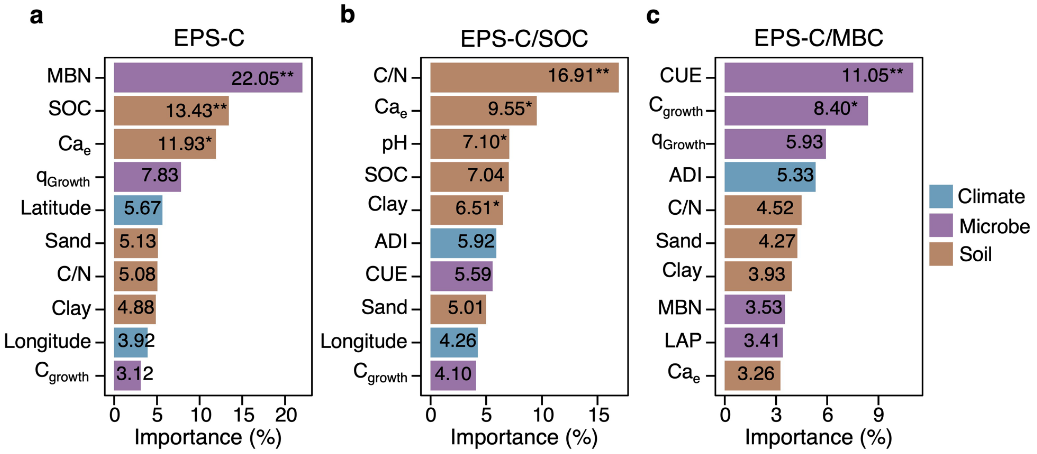

Supplementary Figure 6 | Environmental and microbial drivers of extracellular polymeric substances carbon (EPS-C), EPS-C to soil organic carbon ratio (EPS-C/SOC), EPS-C to microbial biomass carbon ratio (EPS-C/MBC). a Importance ranking of environmental and microbial factors driving EPS-C based on random forest analysis. **b** Importance ranking of predictors for EPS-C/SOC. **c** Importance ranking of predictors for EPS-C/MBC. Significant predictors are indicated: * $p < 0.05$, ** $p < 0.01$, *** $p < 0.001$. MBN, microbial biomass nitrogen; SOC, soil organic carbon; Ca_e, exchangeable calcium (Ca^{2+}); q_{Growth}, microbial growth normalized to microbial biomass carbon; C/N, soil carbon to nitrogen ratio; ADI, aridity index; C_{growth}, microbial growth; CUE, microbial carbon use efficiency; LAP, leucine aminopeptidase.

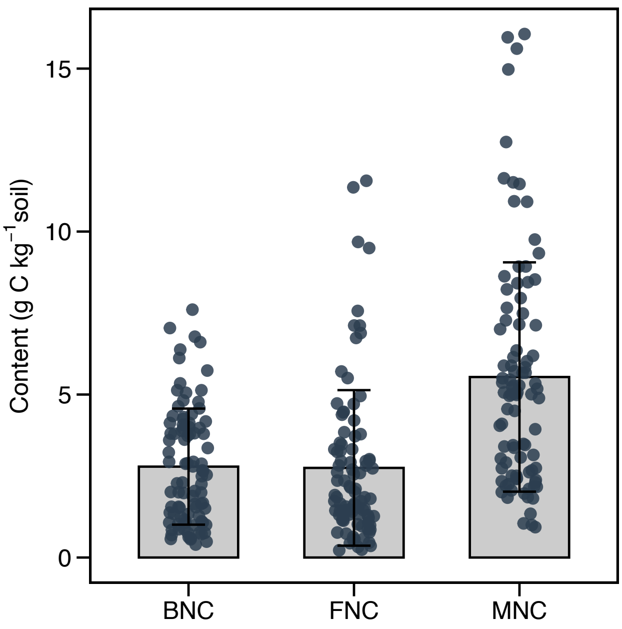

Supplementary Figure 7 | Soil microbial necromass contents across a European transect. Contents of soil bacterial necromass carbon (BNC), soil fungal necromass carbon (FNC) and their sum, soil microbial necromass carbon (MNC) are presented. Bars represent mean values with error bars indicating ± standard deviation (SD). Individual observations are shown as jittered dots.

Supplementary Tables

Supplementary Table 1 | Previously published quantitative data on extracellular polymeric substances (EPS) extracted using the cation exchange resin method.

Reference	Land use type	EPS PS ($\mu g\ g^{-1}$ soil)	EPS PN ($\mu g\ g^{-1}$ soil)	Total EPS ($\mu g\ g^{-1}$ soil)
(Zhang et al., 2023)[11]	Cropland	151.08	86.56	237.64
(Chen et al., 2025)[12]	NA	614.29	117.22	731.52
	NA	121.02	47.67	168.69
	NA	115.72	12.52	128.24
(Redmile-Gordon et al., 2020)[5]	Grass-Grass	346.02	213.17	559.19
	Grass-Arable	339.55	194.56	534.11
	Grass-Fallow	293.99	156.96	450.95
	Arable-Grass	434.87	198.24	633.11
	Arable-Arable	317.79	184.64	502.43
	Arable-Fallow	374.66	175.67	550.33
	Fallow-Grass	381.50	191.27	572.77
	Fallow-Arable	235.55	110.67	346.22
	Fallow-Fallow	286.96	126.43	413.39
(Redmile-Gordon et al., 2014)[10]	Fallow	169.00	43.00	212.00
	Grassland	401.00	163.00	564.00
(Bérard et al., 2020)[13]	Grassland	1120.00	NA	NA
	Cropland	800.00	NA	NA
	Woodland	830.00	NA	NA
	Cropland	743.54	NA	NA
(Kidinda et al., 2023)[6]	Woodland	252.01	52.36	304.36
	Cropland	447.44	273.29	720.74
	Woodland	215.99	60.00	276.00
	Cropland	332.80	194.10	526.90
	Woodland	278.31	80.18	358.49
	Cropland	272.88	145.05	417.93
(Redmile-Gordon et al., 2017)[14]	Grassland	191.33	94.90	286.23

Reference	Land use type	EPS PS ($\mu g\ g^{-1}$ soil)	EPS PN ($\mu g\ g^{-1}$ soil)	Total EPS ($\mu g\ g^{-1}$ soil)
(Shi et al., 2024)[15]	Woodland	158.31	79.28	237.58
(Redmile-Gordon et al., 2015)[16]	Grass-Arable	467.33	177.76	645.08
(Hale et al., 2021)[17]	Grassland	456.41	NA	NA
	Cropland	262.78	NA	NA
(Bettermann et al., 2021)[18]	Shrub land	52.19	NA	NA
	Shrub land	76.30	NA	NA
(Zethof et al., 2020)[19]	Shrub land	130.37	NA	NA
	Shrub land	120.79	NA	NA
(Baumert et al., 2021)[20]	Woodland	158.60	44.90	203.50
	Woodland	160.30	42.30	202.60
	Woodland	98.30	15.70	114.00
(Crouzet et al., 2019)[21]	Cropland	367.95	NA	NA
(Vuko et al., 2020)[22]	Fallow	175.43	78.97	254.40
	Cropland	260.78	133.86	394.64
(Liao et al., 2024)[23]	Woodland	317.20	301.82	619.01
(Liu et al., 2025)[24]	Cropland	471.25	205.19	676.44
	Woodland	691.82	364.28	1056.10
(Peng et al., 2025)[25]	Cropland	297.48	37.23	334.71
(G. Bogar et al., 2025)[26]	Cropland	113.00	NA	NA
(Feng et al., 2026)[27]	Dryland	393.32	114.69	508.01
	Dryland	245.47	43.84	289.31
	Woodland	284.00	100.70	384.70
	Woodland	65.74	50.65	116.39
	Cropland	411.89	104.01	515.90
	Cropland	84.10	54.48	138.58
(Li et al., 2026)[28]	Grassland	489.08	229.30	718.38

Total EPS refers to the sum of EPS polysaccharides and EPS proteins. NA indicates that the data are not available. EPS PN, EPS proteins; EPS PS, EPS polysaccharides.

Supplementary Table 2 | Analysis of variance (ANOVA) results for extracellular polymeric substances (EPS) variables.

Variables	Items	*Df*	*F* value	*p* value
EPS	Bedrock	2	6.69	0.002
	Land use	2	2.77	0.068
EPS polysaccharides	Bedrock	2	9.98	<0.001
	Land use	2	1.06	0.351
EPS proteins	Bedrock	2	0.31	0.732
	Land use	2	6.99	0.002
EPS-C	Bedrock	2	5.93	0.004
	Land use	2	3.21	0.045
EPS-C/SOC	Bedrock	2	16.46	<0.001
	Land use	2	14.68	<0.001
EPS-C/MBC	Bedrock	2	7.38	0.001
	Land use	2	3.96	0.023

EPS-C, extracellular polymeric substances carbon; EPS-C/SOC, extracellular polymeric substances carbon to soil organic carbon ratio; EPS-C/MBC, extracellular polymeric substances carbon to microbial biomass carbon ratio.

Supplementary Table 3 | Linear models explaining microbial biomass carbon (MBC) and microbial biomass nitrogen (MBN).

Response variables	Predictor	Estimate	Std. Error	*t* value	*p* value
MBC	Intercept	1153.60	334.82	3.45	0.001
	pH	-144.48	52.97	-2.73	0.008
	CEC	67.27	25.53	2.64	0.010
	Ca_e	-73.47	68.66	-1.07	0.287
MBN	Intercept	172.76	74.80	2.31	0.023
	pH	-24.78	11.83	-2.09	0.039
	CEC	11.26	5.70	1.97	0.052
	Ca_e	-4.45	15.34	-0.29	0.772

CEC, cation exchange capacity; Ca_e, exchangeable calcium (Ca^{2+}).

Supplementary Table 4 | Two-way analysis of variance (ANOVA) results testing the effects of land use and bedrock on climatic, edaphic, plant, and microbial variables.

Variables	Land use *F* values	Land use *p* values	Bedrock *F* values	Bedrock *p* values	Land use: Bedrock *F* values	Land use: Bedrock *p* values
ADI	6.47	0.002	43.40	<0.001	0.56	0.689
Elevation	1.98	0.144	1.39	0.254	0.57	0.686
Latitude	5.51	0.006	32.30	<0.001	1.23	0.307
Longitude	0.48	0.622	7.68	<0.001	0.48	0.750
MAP	1.24	0.294	5.98	0.004	1.08	0.373
MAT	7.27	0.001	29.23	<0.001	1.49	0.212
PET	2.80	0.067	27.24	<0.001	0.40	0.812
AP	1.74	0.182	1.73	0.183	0.27	0.897
B/F	0.19	0.831	0.73	0.487	1.37	0.250
Bacteria	4.75	0.011	0.30	0.738	0.48	0.752
BG	3.47	0.036	14.21	<0.001	1.31	0.272
BX	1.06	0.351	9.45	<0.001	2.05	0.095
CEL	7.15	0.001	19.45	<0.001	3.06	0.021
C_{growth}	7.46	0.001	4.95	0.009	0.67	0.618
CUE	6.45	0.002	10.13	<0.001	1.21	0.312
Fungi	1.91	0.154	0.31	0.733	0.05	0.995
GNB	4.83	0.010	0.44	0.644	0.35	0.845
GPB	5.21	0.007	0.38	0.684	0.66	0.622
GPB/GNB	0.81	0.451	1.00	0.374	0.29	0.883
LAP	3.86	0.025	18.27	<0.001	1.92	0.114
MBC	4.54	0.013	5.22	0.007	0.47	0.756
MBC/MBN	1.78	0.176	3.17	0.047	0.96	0.435
MBC/MBP	0.74	0.479	10.59	<0.001	0.37	0.829
MBN	2.87	0.062	6.78	0.002	0.42	0.794
MBN/MBP	2.17	0.121	8.23	0.001	0.67	0.618
MBP	1.55	0.218	4.22	0.018	0.61	0.658
MBT	1.21	0.302	0.16	0.854	2.41	0.056
NAG	0.66	0.521	13.91	<0.001	0.91	0.463
NUE	3.76	0.027	9.83	0.000	3.01	0.023
POX	2.25	0.112	1.13	0.327	0.01	1.000
q_{Growth}	5.47	0.006	3.13	0.049	2.21	0.075
Total PLFA	3.61	0.032	0.33	0.722	0.34	0.849
VectorA	1.52	0.225	18.29	<0.001	0.93	0.453
VectorL	16.38	<0.001	4.03	0.021	0.70	0.594
FRB	10.50	<0.001	5.94	0.004	1.23	0.304
Root C	11.12	<0.001	3.36	0.040	1.11	0.357

Variables	Land use F values	Land use p values	Bedrock F values	Bedrock p values	Land use: Bedrock F values	Land use: Bedrock p values
Root C/N	16.60	<0.001	0.72	0.489	3.87	0.006
Root C/P	4.07	0.021	0.21	0.808	1.55	0.196
Root N	13.89	<0.001	0.73	0.486	4.52	0.002
Root N/P	0.72	0.492	0.36	0.700	0.76	0.554
Root P	1.92	0.154	1.79	0.174	2.14	0.085
Al_d	4.91	0.010	0.22	0.800	1.00	0.415
Al_o	6.73	0.002	1.17	0.316	0.65	0.629
C/N	56.02	<0.001	20.38	<0.001	9.43	<0.001
C/P	21.61	<0.001	0.22	0.805	0.57	0.682
Ca_e	6.99	0.002	43.55	<0.001	1.75	0.146
CEC	3.34	0.041	34.48	<0.001	1.30	0.277
Clay	7.45	0.001	13.52	<0.001	1.26	0.291
DOC	6.89	0.002	2.90	0.060	0.33	0.857
Fe_c	2.24	0.113	0.85	0.430	1.74	0.149
Fe_d	0.70	0.501	0.14	0.867	2.51	0.048
Fe_o	8.09	<0.001	2.07	0.133	1.24	0.299
Mg_e	4.73	0.011	2.60	0.080	1.07	0.378
N/P	9.37	<0.001	3.03	0.054	0.71	0.588
NH_4^+	6.31	0.003	2.46	0.092	0.90	0.466
NO_3^-	6.98	0.002	0.30	0.739	2.32	0.063
pH	17.41	<0.001	56.75	<0.001	0.81	0.523
Sand	6.05	0.004	7.30	0.001	1.13	0.350
Silt	2.94	0.058	2.15	0.123	0.60	0.668
SMC	5.47	0.006	12.88	<0.001	0.60	0.666
SOC	6.53	0.002	3.10	0.050	0.27	0.899
TDN	2.68	0.074	0.69	0.506	3.27	0.015
TOP	4.48	0.014	4.67	0.012	3.10	0.020
TP	8.80	0.000	5.38	0.006	1.35	0.257
WHC	3.58	0.032	5.69	0.005	1.08	0.374
TN	1.94	0.147	2.20	0.117	1.58	0.189

For full names of variable abbreviations, see the abbreviations section in the Supplementary Information.

Supplementary Table 5 | Spearman's correlation coefficients and *p* values between environmental and microbial variables and extracellular polymeric substances (EPS), EPS polysaccharides and EPS proteins.

Variables	EPS *r* values	EPS *p* values	EPS PS *r* values	EPS PS *p* values	EPS PN *r* values	EPS PN *p* values
ADI	0.26	0.022	0.24	0.042	0.23	0.045
Elevation	0.23	0.047	0.26	0.024	0.10	0.404
Latitude	0.13	0.250	0.09	0.436	0.19	0.110
Longitude	0.15	0.200	0.11	0.366	0.23	0.048
MAP	0.30	0.008	0.29	0.011	0.21	0.076
MAT	-0.20	0.093	-0.16	0.183	-0.23	0.051
PET	-0.17	0.137	-0.13	0.282	-0.23	0.043
AP	0.12	0.311	0.13	0.264	0.12	0.304
B/F	0.18	0.123	0.13	0.273	0.15	0.207
Bacteria	0.20	0.089	0.14	0.239	0.27	0.021
BG	0.30	0.008	0.31	0.006	0.20	0.088
BX	0.05	0.698	0.10	0.405	-0.02	0.842
CEL	0.35	0.002	0.33	0.004	0.30	0.010
C_{growth}	-0.04	0.716	0.01	0.907	-0.08	0.471
CUE	-0.27	0.019	-0.19	0.098	-0.29	0.011
Fungi	0.10	0.390	0.10	0.382	0.13	0.255
GNB	0.23	0.045	0.18	0.116	0.28	0.015
GPB	0.20	0.089	0.14	0.219	0.25	0.033
GPB/GNB	0.00	0.987	0.00	0.999	-0.04	0.711
LAP	0.19	0.105	0.15	0.188	0.17	0.142
MBC	0.81	0.000	0.75	0.000	0.72	0.000
MBC/MBN	-0.41	0.000	-0.39	0.001	-0.34	0.003
MBC/MBP	0.01	0.954	0.02	0.891	0.03	0.810
MBN	0.82	0.000	0.75	0.000	0.73	0.000
MBN/MBP	0.10	0.385	0.11	0.338	0.09	0.418
MBP	0.33	0.003	0.29	0.011	0.30	0.009
MBT	0.29	0.013	0.22	0.058	0.30	0.009
NAG	0.11	0.328	0.16	0.168	-0.04	0.742
NUE	-0.04	0.706	0.06	0.637	-0.20	0.078
POX	0.09	0.462	0.07	0.560	0.16	0.163
q_{Growth}	-0.54	0.000	-0.45	0.000	-0.53	0.000
Total PLFA	0.16	0.159	0.12	0.303	0.23	0.052
VectorA	-0.04	0.757	-0.07	0.526	0.12	0.299
VectorL	0.26	0.024	0.24	0.039	0.24	0.040
FRB	0.27	0.019	0.26	0.025	0.27	0.020
Root C	-0.04	0.757	0.05	0.648	-0.19	0.094
Root C/N	-0.32	0.006	-0.25	0.033	-0.37	0.001

Variables	EPS *r* values	EPS *p* values	EPS PS *r* values	EPS PS *p* values	EPS PN *r* values	EPS PN *p* values
Root C/P	-0.12	0.314	-0.06	0.602	-0.22	0.054
Root N	0.38	0.001	0.33	0.003	0.38	0.001
Root N/P	0.16	0.172	0.17	0.143	0.07	0.547
Root P	0.13	0.275	0.08	0.474	0.20	0.086
Al_d	0.03	0.818	0.01	0.911	0.07	0.540
Al_o	0.03	0.797	0.03	0.801	0.02	0.888
C/N	-0.19	0.112	-0.10	0.374	-0.29	0.013
C/P	0.10	0.383	0.16	0.164	-0.02	0.890
Ca_e	0.62	0.000	0.59	0.000	0.45	0.000
CEC	0.71	0.000	0.73	0.000	0.45	0.000
Clay	0.48	0.000	0.49	0.000	0.30	0.009
DOC	-0.04	0.716	0.02	0.843	-0.15	0.185
Fe_c	0.06	0.624	0.02	0.865	0.10	0.385
Fe_d	0.06	0.639	0.01	0.957	0.13	0.256
Fe_o	0.06	0.605	0.03	0.807	0.12	0.325
Mg_e	0.51	0.000	0.49	0.000	0.37	0.001
N/P	0.24	0.037	0.27	0.019	0.13	0.249
NH_4^+	0.22	0.055	0.15	0.198	0.36	0.002
NO_3^-	0.29	0.010	0.21	0.074	0.37	0.001
pH	0.15	0.204	0.14	0.216	0.06	0.635
Sand	-0.44	0.000	-0.43	0.000	-0.32	0.005
Silt	0.37	0.001	0.36	0.002	0.32	0.005
SMC	0.40	0.000	0.39	0.000	0.33	0.003
SOC	0.68	0.000	0.68	0.000	0.51	0.000
TDN	0.44	0.000	0.36	0.002	0.48	0.000
TOP	0.64	0.000	0.58	0.000	0.59	0.000
TP	0.52	0.000	0.48	0.000	0.44	0.000
WHC	0.71	0.000	0.64	0.000	0.62	0.000
TN	0.83	0.000	0.79	0.000	0.70	0.000

For full names of variable abbreviations, see the abbreviations section in the Supplementary Information.

Supplementary Table 6 | Variance partitioning analysis (VPA) results showing the relative contributions of land use and bedrock in environmental and microbial variables.

Variables	Bedrock	Land use	Shared	Residuals
ADI	46%	0%	6%	48%
Elevation	1%	1%	2%	97%
Latitude	39%	0%	4%	56%
Longitude	14%	-1%	0%	88%
MAP	10%	0%	0%	90%
MAT	36%	2%	5%	57%
PET	37%	-1%	2%	61%
AP	2%	1%	1%	97%
B/F	-1%	-2%	0%	102%
Bacteria	-1%	6%	2%	93%
BG	22%	3%	0%	75%
BX	15%	1%	-1%	85%
CEL	25%	7%	1%	67%
C_{growth}	7%	7%	5%	81%
CUE	15%	4%	5%	76%
Fungi	-2%	3%	-1%	99%
GNB	-1%	6%	2%	93%
GPB	-1%	7%	2%	93%
GPB/GNB	0%	0%	0%	100%
LAP	26%	4%	0%	70%
MBC	8%	6%	1%	85%
MBC/MBN	5%	1%	0%	94%
MBC/MBP	18%	2%	-3%	82%
MBN	11%	3%	0%	85%
MBN/MBP	14%	5%	-3%	84%
MBP	7%	2%	-1%	92%
MBT	-2%	0%	0%	102%
NAG	23%	0%	-1%	78%
NUE	15%	5%	-1%	81%
POX	0%	2%	1%	97%
q_{Growth}	4%	6%	2%	88%
Total PLFA	-1%	5%	1%	96%
VectorA	28%	0%	0%	72%
VectorL	5%	22%	2%	71%
FRB	8%	11%	4%	76%
Root C	4%	22%	-4%	78%
Root C/N	-1%	24%	0%	77%

Variables	Bedrock	Land use	Shared	Residuals
Root C/P	-2%	6%	1%	95%
Root N	-1%	21%	-1%	81%
Root N/P	-2%	-1%	0%	102%
Root P	2%	0%	2%	96%
Al_d	2%	7%	1%	93%
Al_o	0%	9%	2%	88%
C/N	14%	29%	11%	46%
C/P	-1%	30%	2%	69%
Ca_e	45%	3%	2%	49%
CEC	42%	1%	1%	56%
Clay	20%	6%	3%	71%
DOC	4%	10%	2%	85%
Fe_c	0%	2%	0%	98%
Fe_d	-2%	-1%	0%	103%
Fe_o	2%	14%	0%	85%
Mg_e	3%	5%	2%	90%
N/P	4%	19%	-4%	81%
NH_4^+	3%	11%	-1%	87%
NO_3^-	-1%	9%	2%	90%
pH	49%	9%	4%	38%
Sand	11%	5%	3%	80%
Silt	2%	2%	2%	93%
SMC	20%	2%	5%	73%
SOC	4%	7%	4%	85%
TDN	-1%	4%	-1%	98%
TOP	6%	8%	-2%	88%
TP	8%	19%	-5%	79%
WHC	9%	3%	2%	86%
TN	2%	2%	0%	96%

For full names of variable abbreviations, see the abbreviations section in the Supplementary Information.

Supplementary Table 7 | Mean values and standard deviation (SD) of climatic, edaphic, plant and microbial variables across land use types and bedrock types.

Variables	Carbonate (n = 27)	Sediment (n = 33)	Silicate (n = 32)	Cropland (n = 24)	Grassland (n = 27)	Woodland (n = 41)
ADI	0.59 ± 0.29	0.67 ± 0.12	1.13 ± 0.25	0.68 ± 0.24	0.79 ± 0.34	0.89 ± 0.34
Elevation	405.9 ± 331.6	328.5 ± 271.9	512.6 ± 444.8	303.2 ± 249.9	406.2 ± 396.0	486.8 ± 383.5
Latitude	42.84 ± 4.65	46.37 ± 4.86	57.97 ± 10.49	46.58 ± 6.82	47.67 ± 8.01	52.12 ± 11.44
Longitude	3.72 ± 10.58	13.78 ± 13.55	15.07 ± 10.03	9.92 ± 12.53	10.47 ± 12.24	12.60 ± 12.72
MAP	648.3 ± 178.8	619.1 ± 61.1	790.7 ± 301.6	632.8 ± 124.4	720.3 ± 242.3	697.6 ± 240.8
MAT	12.21 ± 3.52	9.87 ± 3.49	3.91 ± 5.12	10.43 ± 3.85	9.39 ± 4.58	6.75 ± 6.14
PET	3.36 ± 0.86	2.62 ± 0.60	1.86 ± 0.75	2.82 ± 0.88	2.64 ± 0.88	2.38 ± 1.00
AP	1810.3 ± 1119.1	1238.1 ± 934.9	1908.9 ± 1880.0	1181.3 ± 751.2	1809.9 ± 1326.8	1795.1 ± 1680.4
B/F	4.21 ± 2.31	3.94 ± 2.10	4.67 ± 2.40	4.11 ± 2.01	4.18 ± 2.62	4.43 ± 2.20
Bacteria	0.30 ± 0.39	0.26 ± 0.29	0.39 ± 0.45	0.19 ± 0.11	0.23 ± 0.23	0.45 ± 0.51
BG	1799.0 ± 1745.3	543.3 ± 638.1	534.1 ± 339.8	1294.7 ± 1695.7	996.1 ± 1139.6	625.1 ± 691.5
BX	442.6 ± 471.4	105.9 ± 111.2	240.0 ± 281.4	237.7 ± 339.0	321.1 ± 458.1	213.4 ± 225.2
CEL	289.2 ± 229.7	98.3 ± 85.9	100.1 ± 81.8	260.6 ± 238.3	163.3 ± 150.7	105.1 ± 95.9
C_{growth}	587.5 ± 479.4	368.2 ± 496.1	913.1 ± 709.6	362.0 ± 256.1	475.9 ± 466.1	870.6 ± 749.0
CUE	0.38 ± 0.18	0.28 ± 0.17	0.49 ± 0.16	0.34 ± 0.17	0.31 ± 0.18	0.45 ± 0.19
Fungi	0.10 ± 0.10	0.09 ± 0.11	0.09 ± 0.08	0.07 ± 0.07	0.08 ± 0.09	0.11 ± 0.11
GNB	0.15 ± 0.19	0.13 ± 0.15	0.20 ± 0.21	0.09 ± 0.07	0.12 ± 0.12	0.22 ± 0.24
GPB	0.13 ± 0.18	0.12 ± 0.13	0.18 ± 0.20	0.08 ± 0.05	0.11 ± 0.10	0.20 ± 0.22
GPB/GNB	0.90 ± 0.28	0.99 ± 0.28	0.98 ± 0.20	0.94 ± 0.28	0.92 ± 0.29	1.00 ± 0.21
LAP	167.4 ± 175.8	35.6 ± 32.3	38.6 ± 25.2	74.7 ± 83.6	114.4 ± 159.3	50.0 ± 85.6

Variables	Carbonate (n = 27)	Sediment (n = 33)	Silicate (n = 32)	Cropland (n = 24)	Grassland (n = 27)	Woodland (n = 41)
MBC	1034.1 ± 594.1	618.9 ± 345.4	985.2 ± 641.0	599.5 ± 334.4	1025.6 ± 561.4	921.7 ± 626.0
MBC/MBN	5.44 ± 2.07	6.82 ± 2.83	7.05 ± 2.55	6.31 ± 2.98	5.87 ± 2.05	7.02 ± 2.63
MBC/MBP	110.6 ± 184.5	278.7 ± 261.6	425.3 ± 347.1	319.5 ± 335.6	304.6 ± 337.0	241.5 ± 255.0
MBN	215.6 ± 152.5	99.0 ± 63.0	163.9 ± 139.4	108.8 ± 85.5	189.6 ± 112.8	161.1 ± 154.5
MBN/MBP	19.18 ± 34.46	44.47 ± 46.32	64.23 ± 58.43	53.97 ± 65.10	52.23 ± 58.18	32.57 ± 31.65
MBP	21.46 ± 20.37	9.24 ± 11.61	11.06 ± 20.20	8.33 ± 9.68	13.66 ± 16.54	16.32 ± 22.37
MBT	38.66 ± 18.02	38.97 ± 24.60	34.66 ± 26.23	38.86 ± 22.04	42.00 ± 23.90	33.46 ± 23.56
NAG	671.8 ± 633.3	213.4 ± 210.3	22.8 ± 153.6	302.4 ± 295.6	418.0 ± 589.5	335.8 ± 363.1
NUE	0.82 ± 0.23	0.55 ± 0.28	0.68 ± 0.23	0.77 ± 0.23	0.59 ± 0.30	0.67 ± 0.26
POX	0.86 ± 3.53	1.87 ± 2.23	2.50 ± 4.55	0.73 ± 1.50	1.47 ± 1.45	2.62 ± 5.00
q_{Growth}	0.69 ± 0.45	0.68 ± 0.66	1.14 ± 0.97	0.80 ± 0.70	0.51 ± 0.40	1.08 ± 0.90
Total PLFA	0.33 ± 0.31	0.35 ± 0.38	0.45 ± 0.44	0.25 ± 0.17	0.32 ± 0.27	0.49 ± 0.49
VectorA	49.31 ± 2.95	52.74 ± 2.31	53.24 ± 2.64	51.55 ± 2.73	51.42 ± 3.05	52.44 ± 3.31
VectorL	1.49 ± 0.10	1.44 ± 0.12	1.41 ± 0.10	1.52 ± 0.11	1.47 ± 0.08	1.39 ± 0.10
FRB	1.88 ± 2.11	2.45 ± 1.69	4.67 ± 4.30	0.86 ± 1.43	3.57 ± 4.00	4.00 ± 2.70
Root C	402.7 ± 39.8 (n=26)	397.0 ± 74.2 (n=31)	378.9 ± 73.8 (n=32)	368.0 ± 68.4 (n=22)	363.1 ± 61.4 (n=26)	423.5 ± 53.7 (n=41)
Root C/N	46.71 ± 14.25 (n=26)	51.48 ± 22.75 (n=31)	54.77 ± 30.83 (n=32)	44.06 ± 23.11 (n=22)	37.19 ± 15.41 (n=26)	64.06 ± 22.82 (n=41)
Root C/P	629.0 ± 341.5 (n=22)	541.7 ± 366.4 (n=27)	635.0 ± 367.2 (n=31)	477.3 ± 273.5 (n=16)	507.0 ± 389.4 (n=25)	713.8 ± 340.6 (n=39)
Root N	9.36 ± 2.82 (n=26)	8.78 ± 3.14 (n=31)	8.95 ± 4.49 (n=32)	9.55 ± 3.10 (n=22)	11.20 ± 4.25 (n=26)	7.34 ± 2.37 (n=41)

Variables	Carbonate (n = 27)	Sediment (n = 33)	Silicate (n = 32)	Cropland (n = 24)	Grassland (n = 27)	Woodland (n = 41)
Root N/P	13.42 ± 5.30 (n=22)	11.80 ± 9.44 (n=27)	12.17 ± 6.50 (n=30)	12.27 ± 7.91 (n=16)	13.85 ± 9.27 (n=24)	11.54 ± 5.55 (n=39)
Root P	0.82 ± 0.44 (n=22)	1.00 ± 0.57 (n=26)	0.74 ± 0.35 (n=30)	0.96 ± 0.57 (n=15)	0.94 ± 0.40 (n=24)	0.75 ± 0.45 (n=39)
Al_d	1081.3 ± 1191.4 (n=27)	1122.0 ± 893.2 (n=33)	1357.5 ± 914.0 (n=31)	919.5 ± 1160.8 (n=24)	902.7 ± 701.3 (n=27)	1546.6 ± 960.6 (n=40)
Al_o	1399.0 ± 1259.3 (n=27)	1524.4 ± 1238.3 (n=33)	2098.6 ± 1580.5 (n=31)	1118.7 ± 1259.4 (n=24)	1351.0 ± 1100.4 (n=27)	2245.2 ± 1453.3 (n=40)
C/N	11.68 ± 2.93	11.77 ± 3.03	17.83 ± 7.30	10.58 ± 2.69	10.63 ± 1.89	17.89 ± 6.08
C/P	58.79 ± 44.55	60.90 ± 50.00	78.35 ± 66.76	30.90 ± 24.17	44.76 ± 28.61	101.3 ± 61.11
Ca_e	8.85 ± 4.13	3.31 ± 2.82	1.80 ± 2.08	5.74 ± 3.86	5.11 ± 4.47	3.17 ± 4.00
CEC	24.40 ± 10.24	11.30 ± 7.32	8.24 ± 5.35	16.70 ± 9.92	15.09 ± 11.14	11.89 ± 9.58
Clay	24.37 ± 9.74	15.93 ± 9.34	10.88 ± 9.80	22.18 ± 12.07	17.10 ± 10.37	13.11 ± 9.39
DOC	186.8 ± 103.1	126.1 ± 83.9	171.5 ± 92.6	117.6 ± 86.3	140.4 ± 67.1	197.1 ± 103.7
Fe_c	8173.4 ± 7489.2 (n=27)	6417.0 ± 3785.7 (n=33)	6010.4 ± 7063.0 (n=31)	9015.5 ± 9061.6 (n=24)	6310.9 ± 5277.3 (n=27)	5800.0 ± 4271.6 (n=40)
Fe_d	11004.6 ± 8546.8 (n=27)	10423.1 ± 5386.6 (n=33)	10007.6 ± 6914.7 (n=31)	11469.1 ± 9471.7 (n=24)	9275.3 ± 5929.3 (n=27)	10640.8 ± 5628.2 (n=40)
Fe_o	2831.3 ± 3124.2 (n=27)	4006.1 ± 3045.7 (n=33)	3997.3 ± 1926.5 (n=31)	2453.6 ± 1594.2 (n=24)	2964.5 ± 2429.8 (n=27)	4840.8 ± 3089.6 (n=40)
Mg_e	0.37 ± 0.21	0.29 ± 0.22	0.22 ± 0.21	0.39 ± 0.20	0.29 ± 0.21	0.23 ± 0.22
N/P	4.81 ± 2.78	4.89 ± 3.46	4.04 ± 2.60	2.89 ± 2.03	4.16 ± 2.49	5.82 ± 3.22
NH_4^+	1.63 ± 2.89	3.14 ± 3.42	2.90 ± 2.82	1.05 ± 0.66	2.40 ± 2.74	3.67 ± 3.76

Variables	Carbonate (n = 27)	Sediment (n = 33)	Silicate (n = 32)	Cropland (n = 24)	Grassland (n = 27)	Woodland (n = 41)
NO_3^-	9.60 ± 10.27	10.23 ± 7.87	7.11 ± 8.49	12.14 ± 8.30	11.50 ± 9.87	5.42 ± 7.22
pH	7.90 ± 1.14	5.97 ± 1.16	5.26 ± 0.72	7.04 ± 1.26	6.53 ± 1.36	5.69 ± 1.47
Sand	30.73 ± 18.10	43.05 ± 25.01	54.93 ± 22.41	33.70 ± 23.48	39.69 ± 22.87	51.89 ± 22.82
Silt	44.91 ± 14.72	41.04 ± 18.60	34.20 ± 17.19	44.12 ± 18.32	43.21 ± 16.80	35.01 ± 16.48
SMC	0.18 ± 0.11	0.17 ± 0.08	0.30 ± 0.13	0.16 ± 0.07	0.21 ± 0.13	0.25 ± 0.13
SOC	30.88 ± 22.74	22.55 ± 15.50	39.33 ± 25.37	19.08 ± 0.48	29.62 ± 20.53	38.51 ± 25.91
TDN	26.19 ± 20.98	22.16 ± 11.46	24.73 ± 23.04	25.03 ± 17.56	30.05 ± 23.64	19.93 ± 15.03
TOP	306.9 ± 232.9	239.5 ± 166.5	370.6 ± 250.1	279.6 ± 183.6	400.3 ± 245.4	256.9 ± 213.3
TP	0.62 ± 0.37	0.51 ± 0.33	0.69 ± 0.40	0.75 ± 0.37	0.72 ± 0.42	0.44 ± 0.26
WHC	0.66 ± 0.25	0.51 ± 0.16	0.68 ± 0.21	0.52 ± 0.18	0.65 ± 0.22	0.64 ± 0.23
TN	2.65 ± 1.81	1.90 ± 0.99	2.53 ± 1.65	1.87 ± 1.02	2.68 ± 1.46	2.38 ± 1.76

Sample sizes differing from those indicated in the column headers are provided for individual variables. For full names of variable abbreviations, see the abbreviations section in the Supplementary Information.

Supplementary Table 8 | Effects of climate, plant, soil, and microbial factors on extracellular polymeric substances (EPS), evaluated using *p* and *F* values from the general linear model.

Variables	*Df*	*F* value	*p* value
Latitude	1	0.10	0.755
Longitude	1	1.70	0.196
Elevation	1	8.89	0.004
MAT	1	1.02	0.316
MAP	1	12.32	<0.001
PET	1	1.81	0.182
ADI	1	4.63	0.034
FRB	1	8.12	0.005
Root C	1	0.20	0.655
Root N	1	11.47	0.001
Root P	1	1.36	0.247
Root C/N	1	9.85	0.002
Root C/P	1	1.36	0.246
Root N/P	1	0.25	0.620
MBC	1	124.90	<0.001
MBN	1	135.26	<0.001
MBP	1	38.64	<0.001
MBC/MBN	1	13.81	<0.001
MBC/MBP	1	0.81	0.369
MBN/MBP	1	0.02	0.900
CUE	1	5.35	0.023
NUE	1	0.04	0.847
C_{growth}	1	0.30	0.586
q_{Growth}	1	19.84	<0.001
MBT	1	3.72	0.057
VectorL	1	2.70	0.104
VectorA	1	0.52	0.475
GPB	1	2.93	0.090
GNB	1	4.13	0.045
Fungi	1	2.04	0.157
Bacteria	1	2.90	0.092
Total PLFA	1	1.56	0.216
B/F	1	0.25	0.617
GPB/GNB	1	0.01	0.946
BG	1	0.01	0.918
NAG	1	1.07	0.304
LAP	1	0.06	0.803
AP	1	4.79	0.031

Variables	*Df*	*F* value	*p* value
CEL	1	2.53	0.115
BX	1	0.53	0.471
POX	1	0.67	0.415
SOC	1	46.54	<0.001
TN	1	128.05	<0.001
TP	1	26.83	<0.001
C/N	1	5.04	0.027
C/P	1	0.21	0.650
N/P	1	2.40	0.125
NH_4^+	1	0.43	0.514
NO_3^-	1	11.85	<0.001
TDN	1	18.84	<0.001
DOC	1	0.21	0.650
TOP	1	44.70	<0.001
WHC	1	73.77	<0.001
CEC	1	77.62	<0.001
SMC	1	16.10	<0.001
Sand	1	15.14	<0.001
Silt	1	7.25	0.008
Clay	1	17.01	<0.001
Ca_e	1	61.59	<0.001
Mg_e	1	9.48	0.003
Fe_d	1	1.27	0.263
Fe_c	1	1.68	0.198
Al_o	1	0.27	0.604
Fe_o	1	0.01	0.921
Al_d	1	1.19	0.278

For full names of variable abbreviations, see the abbreviations section in the Supplementary Information.

Supplementary Table 9 | Effects of climate, plant, soil, microbial factors, and land use type on extracellular polymeric substances (EPS), evaluated using *p* and *F* values from the general linear model.

Variables	*Df*	*F* value	*p* value
Latitude	1	0.11	0.744
Land use type	2	2.85	0.063
Latitude:Land use type	2	3.14	0.048
Longitude	1	1.86	0.177
Land use type	2	3.00	0.055
Longitude:Land use type	2	3.10	0.050
Elevation	1	9.39	0.003
Land use type	2	2.96	0.057
Elevation:Land use type	2	1.56	0.215
MAT	1	1.10	0.297
Land use type	2	3.02	0.054
MAT:Land use type	2	2.55	0.084
MAP	1	12.92	<0.001
Land use type	2	2.18	0.119
MAP:Land use type	2	2.00	0.142
PET	1	1.93	0.168
Land use type	2	2.91	0.060
PET:Land use type	2	2.20	0.117
ADI	1	4.74	0.032
Land use type	2	2.76	0.069
ADI:Land use type	2	0.30	0.744
FRB	1	8.56	0.004
Land use type	2	2.25	0.111
FRB:Land use type	2	2.15	0.123
Root C	1	0.20	0.655
Land use type	2	2.08	0.131
Root C:Land use type	2	0.11	0.899
Root N	1	11.69	<0.001
Land use type	2	1.24	0.294
Root N:Land use type	2	1.57	0.215
Root P	1	1.34	0.250
Land use type	2	1.32	0.274
Root P:Land use type	2	0.11	0.900
Root C/N	1	9.83	0.002
Land use type	2	1.65	0.199
Root C/N:Land use type	2	0.28	0.760
Root C/P	1	1.36	0.247

Variables	*Df*	*F* value	*p* value
Land use type	2	1.33	0.271
Root C/P:Land use type	2	0.54	0.585
Root N/P	1	0.25	0.621
Land use type	2	1.22	0.302
Root N/P:Land use type	2	0.70	0.498
MBC	1	129.36	<0.001
Land use type	2	2.41	0.096
MBC:Land use type	2	1.20	0.307
MBN	1	138.74	<0.001
Land use type	2	1.66	0.196
MBN:Land use type	2	1.50	0.230
MBP	1	43.02	<0.001
Land use type	2	3.84	0.025
MBP:Land use type	2	3.27	0.043
MBC/MBN	1	14.03	<0.001
Land use type	2	2.24	0.112
MBC/MBN:Land use type	2	0.47	0.629
MBC/MBP	1	0.92	0.340
Land use type	2	3.07	0.052
MBC/MBP:Land use type	2	4.72	0.011
MBN/MBP	1	0.02	0.893
Land use type	2	3.04	0.053
MBN/MBP:Land use type	2	5.24	0.007
CUE	1	5.58	0.020
Land use type	2	2.39	0.098
CUE:Land use type	2	1.60	0.209
NUE	1	0.04	0.845
Land use type	2	2.75	0.070
NUE:Land use type	2	0.27	0.765
C_{growth}	1	0.32	0.576
Land use type	2	2.92	0.059
C_{growth}:Land use type	2	1.58	0.212
q_{Growth}	1	20.28	<0.001
Land use type	2	1.69	0.192
q_{Growth}:Land use type	2	1.30	0.278
MBT	1	3.85	0.053
Land use type	2	2.45	0.092
MBT:Land use type	2	1.09	0.342
VectorL	1	2.82	0.097
Land use type	2	3.23	0.045
VectorL:Land use type	2	0.74	0.481

Variables	*Df*	*F* value	*p* value
VectorA	1	0.52	0.471
Land use type	2	2.60	0.080
VectorA:Land use type	2	0.06	0.941
GPB	1	3.06	0.084
Land use type	2	3.30	0.042
GPB:Land use type	2	0.75	0.475
GNB	1	4.50	0.037
Land use type	2	3.63	0.031
GNB:Land use type	2	2.36	0.101
Fungi	1	2.20	0.141
Land use type	2	3.01	0.054
Fungi:Land use type	2	2.62	0.079
Bacteria	1	3.10	0.082
Land use type	2	3.37	0.039
Bacteria:Land use type	2	1.63	0.203
Total PLFA	1	1.67	0.200
Land use type	2	3.06	0.052
Total PLFA:Land use type	2	2.21	0.116
B/F	1	0.26	0.610
Land use type	2	0.28	0.069
B/F:Land use type	2	0.82	0.444
GPB/GNB	1	0.01	0.944
Land use type	2	2.83	0.064
GPB/GNB:Land use type	2	2.42	0.095
BG	1	0.01	0.916
Land use type	2	2.78	0.068
BG:Land use type	2	1.43	0.245
NAG	1	1.12	0.292
Land use type	2	2.58	0.082
NAG:Land use type	2	1.69	0.190
LAP	1	0.06	0.801
Land use type	2	2.68	0.075
LAP:Land use type	2	0.12	0.888
AP	1	5.17	0.025
Land use type	2	2.43	0.094
AP:Land use type	2	3.15	0.048
CEL	1	2.70	0.104
Land use type	2	3.28	0.042
CEL:Land use type	2	1.79	0.174
BX	1	0.55	0.462
Land use type	2	2.59	0.081

Variables	*Df*	*F* value	*p* value
BX:Land use type	2	1.12	0.330
POX	1	0.69	0.409
Land use type	2	2.74	0.070
POX:Land use type	2	0.36	0.698
SOC	1	54.88	<0.001
Land use type	2	7.09	0.001
SOC:Land use type	2	2.98	0.056
TN	1	133.06	<0.001
Land use type	2	1.85	0.164
TN:Land use type	2	1.91	0.154
TP	1	29.29	<0.001
Land use type	2	4.65	0.012
TP:Land use type	2	1.46	0.239
C/N	1	5.35	0.023
Land use type	2	3.03	0.054
C/N:Land use type	2	1.75	0.181
C/P	1	0.21	0.646
Land use type	2	2.97	0.056
C/P:Land use type	2	0.12	0.886
N/P	1	2.47	0.120
Land use type	2	2.94	0.058
N/P:Land use type	2	0.31	0.732
NH_4^+	1	0.46	0.499
Land use type	2	2.79	0.067
NH_4^+:Land use type	2	2.43	0.094
NO_3^-	1	12.37	<0.001
Land use type	2	3.12	0.049
NO_3^-:Land use type	2	0.85	0.429
TDN	1	19.22	<0.001
Land use type	2	1.96	0.147
TDN:Land use type	2	0.96	0.389
DOC	1	0.23	0.631
Land use type	2	3.02	0.054
DOC:Land use type	2	4.21	0.018
TOP	1	43.82	<0.001
Land use type	2	0.91	0.408
TOP:Land use type	2	0.20	0.816
WHC	1	75.77	<0.001
Land use type	2	2.97	0.056
WHC:Land use type	2	0.25	0.779
CEC	1	87.25	<0.001

Variables	*Df*	*F* value	*p* value
Land use type	2	7.08	0.001
CEC:Land use type	2	0.51	0.602
SMC	1	16.88	<0.001
Land use type	2	3.47	0.036
SMC:Land use type	3	0.70	0.499
Sand	1	15.96	<0.001
Land use type	2	4.01	0.022
Sand:Land use type	2	0.42	0.658
Silt	1	7.39	0.008
Land use type	2	2.71	0.072
Silt:Land use type	2	0.12	0.886
Clay	1	18.34	<0.001
Land use type	2	5.42	0.006
Clay:Land use type	2	0.11	0.899
Ca_e	1	68.92	<0.001
Land use type	2	6.68	0.002
Ca_e:Land use type	2	0.68	0.510
Mg_e	1	10.14	0.002
Land use type	2	4.33	0.016
Mg_e:Land use type	2	0.80	0.452
Fe_d	1	1.34	0.251
Land use type	2	3.13	0.049
Fe_d:Land use type	2	1.15	0.322
Fe_c	1	1.75	0.190
Land use type	2	3.17	0.047
Fe_c:Land use type	2	0.65	0.526
Al_o	1	0.31	0.582
Land use type	2	3.03	0.054
Al_o:Land use type	2	4.82	0.010
Fe_o	1	0.01	0.916
Land use type	2	2.86	0.064
Fe_o:Land use type	2	3.98	0.022
Al_d	1	1.35	0.249
Land use type	2	3.38	0.039
Al_d:Land use type	2	4.32	0.016

For full names of variable abbreviations, see the abbreviations section in the Supplementary Information.

Supplementary Table 10 | Effects of climate, plant, soil, microbial factors, and bedrock type on extracellular polymeric substances (EPS), evaluated using *p* and *F* values from the general linear model.

Variables	*Df*	*F* value	*p* value
Latitude	1	0.14	0.715
Bedrock type	2	9.11	<0.001
Latitude:Bedrock type	2	9.59	<0.001
Longitude	1	2.22	0.140
Bedrock type	2	12.07	<0.001
Longitude:Bedrock type	2	3.63	0.031
Elevation	1	10.26	0.002
Bedrock type	2	7.55	<0.001
Elevation:Bedrock type	2	1.34	0.268
MAT	1	1.62	0.207
Bedrock type	2	16.11	<0.001
MAT:Bedrock type	2	12.32	<0.001
MAP	1	14.94	<0.001
Bedrock type	2	10.50	<0.001
MAP:Bedrock type	2	1.05	0.354
PET	1	2.73	0.102
Bedrock type	2	19.46	<0.001
PET:Bedrock type	2	5.34	0.007
ADI	1	7.17	0.009
Bedrock type	2	24.60	<0.001
ADI:Bedrock type	2	1.99	0.143
FRB	1	10.83	0.001
Bedrock type	2	13.04	<0.001
FRB:Bedrock type	2	3.95	0.023
Root C	1	0.23	0.634
Bedrock type	2	7.72	<0.001
Root C:Bedrock type	2	0.12	0.883
Root N	1	13.32	<0.001
Bedrock type	2	7.75	<0.001
Root N:Bedrock type	2	1.28	0.285
Root P	1	1.67	0.201
Bedrock type	2	8.24	<0.001
Root P:Bedrock type	2	2.24	0.114
Root C/N	1	11.38	0.001
Bedrock type	2	7.10	0.001
Root C/N:Bedrock type	2	1.64	0.199
Root C/P	1	1.59	0.211
Bedrock type	2	8.07	<0.001

Variables	*Df*	*F* value	*p* value
Root C/P:Bedrock type	2	0.48	0.619
Root N/P	1	0.29	0.594
Bedrock type	2	7.67	<0.001
Root N/P:Bedrock type	2	0.36	0.696
MBC	1	148.53	<0.001
Bedrock type	2	9.17	<0.001
MBC:Bedrock type	2	1.35	0.266
MBN	1	143.19	<0.001
Bedrock type	2	3.59	0.032
MBN:Bedrock type	2	1.05	0.355
MBP	1	43.20	<0.001
Bedrock type	2	3.26	0.043
MBP:Bedrock type	2	4.06	0.021
MBC/MBN	1	14.69	<0.001
Bedrock type	2	4.28	0.017
MBC/MBN:Bedrock type	2	0.61	0.545
MBC/MBP	1	0.91	0.344
Bedrock type	2	6.20	0.003
MBC/MBP:Bedrock type	2	0.91	0.408
MBN/MBP	1	0.02	0.894
Bedrock type	2	7.08	0.001
MBN/MBP:Bedrock type	2	0.41	0.665
CUE	1	6.81	0.011
Bedrock type	2	9.63	<0.001
CUE:Bedrock type	2	4.66	0.012
NUE	1	0.04	0.836
Bedrock type	2	8.21	<0.001
NUE:Bedrock type	2	0.63	0.534
C_{growth}	1	0.33	0.568
Bedrock type	2	6.50	0.002
C_{growth}:Bedrock type	2	0.10	0.903
q_{Growth}	1	24.86	<0.001
Bedrock type	2	7.99	<0.001
q_{Growth}:Bedrock type	2	5.40	0.006
MBT	1	4.16	0.044
Bedrock type	2	6.68	0.002
MBT:Bedrock type	2	0.69	0.505
VectorL	1	3.00	0.087
Bedrock type	2	5.55	0.005
VectorL:Bedrock type	2	1.40	0.251
VectorA	1	0.58	0.448

Variables	*Df*	*F* value	*p* value
Bedrock type	2	7.35	0.001
VectorA:Bedrock type	2	0.32	0.726
GPB	1	3.30	0.073
Bedrock type	2	7.08	0.001
GPB:Bedrock type	2	0.52	0.598
GNB	1	4.63	0.034
Bedrock type	2	7.03	0.001
GNB:Bedrock type	2	0.43	0.655
Fungi	1	2.26	0.137
Bedrock type	2	6.52	0.002
Fungi:Bedrock type	2	0.33	0.720
Bacteria	1	3.25	0.075
Bedrock type	2	6.92	0.002
Bacteria:Bedrock type	2	0.43	0.653
Total PLFA	1	1.79	0.185
Bedrock type	2	7.29	0.001
Total PLFA:Bedrock type	2	1.47	0.237
B/F	1	0.28	0.597
Bedrock type	2	6.58	0.002
B/F:Bedrock type	2	0.60	0.552
GPB/GNB	1	0.01	0.942
Bedrock type	2	6.78	0.002
GPB/GNB:Bedrock type	2	1.25	0.293
BG	1	0.01	0.907
Bedrock type	2	9.80	<0.001
BG:Bedrock type	2	6.14	0.003
NAG	1	1.20	0.276
Bedrock type	2	6.38	0.003
NAG:Bedrock type	2	1.16	0.320
LAP	1	0.08	0.781
Bedrock type	2	9.25	<0.001
LAP:Bedrock type	2	3.41	0.038
AP	1	6.09	0.016
Bedrock type	2	7.05	0.001
AP:Bedrock type	2	7.23	0.001
CEL	1	2.80	0.098
Bedrock type	2	5.33	0.007
CEL:Bedrock type	2	1.47	0.235
BX	1	0.70	0.407
Bedrock type	2	7.76	<0.001
BX:Bedrock type	2	8.74	<0.001

Variables	*Df*	*F* value	*p* value
POX	1	0.77	0.383
Bedrock type	2	7.52	<0.001
POX:Bedrock type	2	1.13	0.327
SOC	1	58.74	<0.001
Bedrock type	2	12.84	<0.001
SOC:Bedrock type	2	0.96	0.387
TN	1	150.75	<0.001
Bedrock type	2	9.48	<0.001
TN:Bedrock type	2	0.50	0.611
TP	1	33.07	<0.001
Bedrock type	2	8.89	<0.001
TP:Bedrock type	2	3.56	0.033
C/N	1	5.49	0.021
Bedrock type	2	5.88	0.004
C/N:Bedrock type	2	0.13	0.880
C/P	1	0.23	0.632
Bedrock type	2	6.70	0.002
C/P:Bedrock type	2	0.23	0.796
N/P	1	2.69	0.105
Bedrock type	2	6.58	0.002
N/P:Bedrock type	2	0.85	0.432
NH_4^+	1	0.50	0.481
Bedrock type	2	7.79	<0.001
NH_4^+:Bedrock type	2	1.69	0.190
NO_3^-	1	14.09	<0.001
Bedrock type	2	7.68	<0.001
NO_3^-:Bedrock type	2	2.83	0.065
TDN	1	20.97	<0.001
Bedrock type	2	6.68	0.002
TDN:Bedrock type	2	0.41	0.662
DOC	1	0.24	0.623
Bedrock type	2	6.85	0.002
DOC:Bedrock type	2	3.03	0.053
TOP	1	54.21	<0.001
Bedrock type	2	11.09	<0.001
TOP:Bedrock type	2	0.48	0.618
WHC	1	86.16	<0.001
Bedrock type	2	9.07	<0.001
WHC:Bedrock type	2	0.49	0.616
CEC	1	81.46	<0.001
Bedrock type	2	3.03	0.053

Variables	*Df*	*F* value	*p* value
CEC:Bedrock type	2	1.20	0.306
SMC	1	21.97	<0.001
Bedrock type	2	17.92	<0.001
SMC:Bedrock type	2	0.48	0.622
Sand	1	16.97	<0.001
Bedrock type	2	4.17	0.019
Sand:Bedrock type	2	3.28	0.043
Silt	1	8.58	0.004
Bedrock type	2	6.00	0.004
Silt:Bedrock type	2	4.22	0.018
Clay	1	17.32	<0.001
Bedrock type	2	2.77	0.068
Clay:Bedrock type	2	0.07	0.934
Ca_e	1	63.94	<0.001
Bedrock type	2	2.89	0.061
Ca_e:Bedrock type	2	0.82	0.443
Mg_e	1	10.57	0.002
Bedrock type	2	5.19	0.007
Mg_e:Bedrock type	2	1.97	0.146
Fe_d	1	1.42	0.237
Bedrock type	2	6.31	0.003
Fe_d:Bedrock type	2	0.82	0.444
Fe_c	1	1.89	0.173
Bedrock type	2	6.04	0.004
Fe_c:Bedrock type	2	1.57	0.213
Al_o	1	0.33	0.568
Bedrock type	2	7.34	0.001
Al_o:Bedrock type	2	4.23	0.018
Fe_o	1	0.01	0.916
Bedrock type	2	6.54	0.002
Fe_o:Bedrock type	2	0.30	0.756
Al_d	1	1.39	0.241
Bedrock type	2	7.08	0.001
Al_d:Bedrock type	2	2.38	0.099

For full names of variable abbreviations, see the abbreviations section in the Supplementary Information.

Supplementary Table 11 | Effects of climate, plant, soil, and microbial factors on extracellular polymeric substances (EPS) polysaccharides, evaluated using *p* and *F* values from the general linear model.

Variables	*Df*	*F* value	*p* value
Latitude	1	0.55	0.459
Longitude	1	0.52	0.475
Elevation	1	10.71	0.002
MAT	1	0.36	0.550
MAP	1	11.91	<0.001
PET	1	0.40	0.527
ADI	1	3.28	0.073
FRB	1	5.17	0.025
Root C	1	0.19	0.666
Root N	1	5.61	0.020
Root P	1	0.88	0.352
Root C/N	1	5.32	0.023
Root C/P	1	0.55	0.461
Root N/P	1	0.20	0.659
MBC	1	95.30	<0.001
MBN	1	112.41	<0.001
MBP	1	41.30	<0.001
MBC/MBN	1	11.28	0.001
MBC/MBP	1	1.65	0.203
MBN/MBP	1	0.37	0.546
CUE	1	3.59	0.061
NUE	1	0.39	0.536
C_{growth}	1	0.75	0.390
q_{Growth}	1	12.70	<0.001
MBT	1	1.95	0.166
VectorL	1	1.38	0.244
VectorA	1	2.24	0.138
GPB	1	2.12	0.149
GNB	1	3.28	0.074
Fungi	1	1.84	0.178
Bacteria	1	2.12	0.149
Total PLFA	1	0.97	0.327
B/F	1	0.07	0.793
GPB/GNB	1	0.01	0.931
BG	1	0.18	0.670
NAG	1	1.98	0.163
LAP	1	0.31	0.578
AP	1	5.03	0.027

Variables	*Df*	*F* value	*p* value
CEL	1	2.83	0.096
BX	1	0.61	0.437
POX	1	0.62	0.435
SOC	1	48.32	<0.001
TN	1	94.64	<0.001
TP	1	17.73	<0.001
C/N	1	2.54	0.115
C/P	1	1.30	0.258
N/P	1	3.88	0.052
NH_4^+	1	0.02	0.904
NO_3^-	1	6.40	0.013
TDN	1	11.96	<0.001
DOC	1	1.06	0.306
TOP	1	30.84	<0.001
WHC	1	60.23	<0.001
CEC	1	101.51	<0.001
SMC	1	14.84	<0.001
Sand	1	14.14	<0.001
Silt	1	5.31	0.024
Clay	1	20.81	<0.001
Ca_e	1	76.14	<0.001
Mg_e	1	9.12	0.003
Fe_d	1	1.21	0.274
Fe_c	1	1.98	0.163
Al_o	1	0.24	0.626
Fe_o	1	0.17	0.685
Al_d	1	1.09	0.301

For full names of variable abbreviations, see the abbreviations section in the Supplementary Information.

Supplementary Table 12 | Effects of climate, plant, soil, microbial factors, and land use type on extracellular polymeric substances (EPS) polysaccharides, evaluated using *p* and *F* values from the general linear model.

Variables	*Df*	*F* value	*p* value
Latitude	1	0.56	0.455
Land use type	2	1.10	0.337
Latitude:Land use type	2	1.76	0.179
Longitude	1	0.54	0.464
Land use type	2	1.10	0.337
Longitude:Land use type	2	3.10	0.050
Elevation	1	11.22	0.001
Land use type	2	1.04	0.357
Elevation:Land use type	2	3.07	0.051
MAT	1	0.36	0.549
Land use type	2	1.04	0.357
MAT:Land use type	2	1.17	0.317
MAP	1	12.23	<0.001
Land use type	2	0.60	0.554
MAP:Land use type	2	2.61	0.079
PET	1	0.41	0.526
Land use type	2	1.03	0.362
PET:Land use type	2	1.32	0.272
ADI	1	3.22	0.076
Land use type	2	0.92	0.404
ADI:Land use type	2	0.28	0.757
FRB	1	5.41	0.022
Land use type	2	0.64	0.531
FRB:Land use type	2	3.44	0.037
Root C	1	0.18	0.670
Land use type	2	0.91	0.406
Root C:Land use type	2	0.08	0.921
Root N	1	5.66	0.020
Land use type	2	0.68	0.512
Root N:Land use type	2	1.73	0.184
Root P	1	0.84	0.363
Land use type	2	0.23	0.795
Root P:Land use type	2	0.09	0.916
Root C/N	1	5.23	0.025
Land use type	2	0.94	0.397
Root C/N:Land use type	2	0.30	0.746
Root C/P	1	0.53	0.469
Land use type	2	0.26	0.770

Variables	*Df*	*F* value	*p* value
Root C/P:Land use type	2	0.39	0.681
Root N/P	1	0.19	0.664
Land use type	2	0.20	0.818
Root N/P:Land use type	2	0.58	0.564
MBC	1	95.65	<0.001
Land use type	2	0.92	0.402
MBC:Land use type	2	1.25	0.293
MBN	1	111.58	<0.001
Land use type	2	0.29	0.752
MBN:Land use type	2	1.39	0.256
MBP	1	42.96	<0.001
Land use type	2	1.24	0.295
MBP:Land use type	2	2.57	0.082
MBC/MBN	1	11.11	0.001
Land use type	2	0.92	0.403
MBC/MBN:Land use type	2	0.39	0.679
MBC/MBP	1	1.76	0.188
Land use type	2	1.15	0.323
MBC/MBP:Land use type	2	3.94	0.023
MBN/MBP	1	0.40	0.528
Land use type	2	1.18	0.314
MBN/MBP:Land use type	2	5.14	0.008
CUE	1	3.67	0.059
Land use type	2	1.07	0.348
CUE:Land use type	2	1.87	0.160
NUE	1	0.38	0.538
Land use type	2	1.35	0.264
NUE:Land use type	2	0.18	0.835
C_{growth}	1	0.76	0.385
Land use type	2	1.12	0.333
Cgrowth:Land use type	2	2.01	0.140
q_{Growth}	1	12.76	<0.001
Land use type	2	0.86	0.429
q_{Growth}:Land use type	2	1.39	0.255
MBT	1	1.96	0.165
Land use type	2	0.96	0.388
MBT:Land use type	2	1.29	0.280
VectorL	1	1.38	0.243
Land use type	2	1.51	0.227
VectorL:Land use type	2	0.60	0.554
VectorA	1	2.20	0.142

Variables	*Df*	*F* value	*p* value
Land use type	2	1.00	0.371
VectorA:Land use type	2	0.21	0.813
GPB	1	2.14	0.147
Land use type	2	1.18	0.313
GPB:Land use type	2	1.19	0.310
GNB	1	3.49	0.065
Land use type	2	1.34	0.268
GNB:Land use type	2	3.52	0.034
Fungi	1	1.92	0.169
Land use type	2	1.09	0.340
Fungi:Land use type	2	2.91	0.060
Bacteria	1	2.19	0.143
Land use type	2	1.22	0.301
Bacteria:Land use type	2	2.38	0.098
Total PLFA	1	1.02	0.316
Land use type	2	1.11	0.334
Total PLFA:Land use type	2	3.00	0.055
B/F	1	0.07	0.794
Land use type	2	1.04	0.360
B/F:Land use type	2	0.46	0.631
GPB/GNB	1	0.01	0.930
Land use type	2	1.08	0.346
GPB/GNB:Land use type	2	2.34	0.103
BG	1	0.19	0.667
Land use type	2	1.12	0.330
BG:Land use type	2	1.79	0.173
NAG	1	2.01	0.160
Land use type	2	0.89	0.414
NAG:Land use type	2	1.98	0.145
LAP	1	0.31	0.582
Land use type	2	0.93	0.398
LAP:Land use type	2	0.13	0.875
AP	1	5.28	0.024
Land use type	2	0.74	0.481
AP:Land use type	2	3.48	0.035
CEL	1	2.91	0.092
Land use type	2	1.60	0.208
CEL:Land use type	2	1.72	0.187
BX	1	0.61	0.436
Land use type	2	0.96	0.389
BX:Land use type	2	1.31	0.275

Variables	*Df*	*F* value	*p* value
POX	1	0.61	0.437
Land use type	2	1.01	0.370
POX:Land use type	2	0.65	0.523
SOC	1	52.69	<0.001
Land use type	2	3.55	0.033
SOC:Land use type	2	2.53	0.086
TN	1	95.00	<0.001
Land use type	2	0.16	0.855
TN:Land use type	2	2.02	0.140
TP	1	18.64	<0.001
Land use type	2	2.80	0.066
TP:Land use type	2	1.52	0.224
C/N	1	2.67	0.106
Land use type	2	1.72	0.185
C/N:Land use type	2	2.56	0.083
C/P	1	1.28	0.261
Land use type	2	1.34	0.269
C/P:Land use type	2	0.17	0.847
N/P	1	3.83	0.054
Land use type	2	1.11	0.333
N/P:Land use type	2	0.27	0.767
NH_4^+	1	0.02	0.903
Land use type	2	1.07	0.347
NH_4^+:Land use type	2	2.22	0.115
NO_3^-	1	6.44	0.013
Land use type	2	1.51	0.227
NO_3^-:Land use type	2	0.71	0.493
TDN	1	11.96	<0.001
Land use type	2	0.82	0.443
TDN:Land use type	2	1.16	0.317
DOC	1	1.14	0.289
Land use type	2	1.15	0.321
DOC:Land use type	2	4.14	0.019
TOP	1	30.15	<0.001
Land use type	2	0.50	0.611
TOP:Land use type	2	0.49	0.614
WHC	1	59.31	<0.001
Land use type	2	0.84	0.436
WHC:Land use type	2	0.48	0.622
CEC	1	109.79	<0.001
Land use type	2	5.50	0.006

Variables	*Df*	*F* value	*p* value
CEC:Land use type	2	0.17	0.844
SMC	1	15.09	<0.001
Land use type	2	1.23	0.296
SMC:Land use type	2	1.53	0.223
Sand	1	14.53	<0.001
Land use type	2	2.44	0.093
Sand:Land use type	2	0.81	0.450
Silt	1	5.24	0.025
Land use type	2	1.21	0.302
Silt:Land use type	2	0.22	0.804
Clay	1	21.87	<0.001
Land use type	2	3.94	0.023
Clay:Land use type	2	0.33	0.720
Ca_e	1	83.68	<0.001
Land use type	2	5.81	0.004
Ca_e:Land use type	2	0.65	0.524
Mg_e	1	9.37	0.003
Land use type	2	2.39	0.098
Mg_e:Land use type	2	0.85	0.432
Fe_d	1	1.24	0.268
Land use type	2	1.29	0.280
Fe_d:Land use type	2	1.76	0.178
Fe_c	1	2.01	0.160
Land use type	2	1.46	0.238
Fe_c:Land use type	2	1.24	0.296
Al_o	1	0.26	0.614
Land use type	2	1.11	0.335
Al_o:Land use type	2	4.07	0.021
Fe_o	1	0.17	0.677
Land use type	2	1.12	0.330
Fe_o:Land use type	2	3.27	0.043
Al_d	1	1.16	0.285
Land use type	2	1.25	0.291
Al_d:Land use type	2	3.72	0.028

For full names of variable abbreviations, see the abbreviations section in the Supplementary Information.

Supplementary Table 13 | Effects of climate, plant, soil, microbial factors, and bedrock type on extracellular polymeric substances (EPS) polysaccharides, evaluated using *p* and *F* values from the general linear model.

Variables	*Df*	*F* value	*p* value
Latitude	1	0.78	0.381
Bedrock type	2	11.87	<0.001
Latitude:Bedrock type	2	8.20	<0.001
Longitude	1	0.72	0.398
Bedrock type	2	15.51	<0.001
Longitude:Bedrock type	2	4.35	0.016
Elevation	1	13.35	<0.001
Bedrock type	2	11.53	<0.001
Elevation:Bedrock type	2	1.52	0.225
MAT	1	0.60	0.442
Bedrock type	2	19.86	<0.001
MAT:Bedrock type	2	11.67	<0.001
MAP	1	15.79	<0.001
Bedrock type	2	15.04	<0.001
MAP:Bedrock type	2	1.64	0.201
PET	1	0.62	0.433
Bedrock type	2	21.26	<0.001
PET:Bedrock type	2	5.17	0.008
ADI	1	5.40	0.022
Bedrock type	2	29.16	<0.001
ADI:Bedrock type	2	1.93	0.151
FRB	1	7.38	0.008
Bedrock type	2	16.69	<0.001
FRB:Bedrock type	2	4.60	0.013
Root C	1	0.23	0.635
Bedrock type	2	10.61	<0.001
Root C:Bedrock type	2	0.45	0.639
Root N	1	7.11	0.009
Bedrock type	2	11.16	<0.001
Root N:Bedrock type	2	2.49	0.089
Root P	1	1.14	0.289
Bedrock type	2	11.21	<0.001
Root P:Bedrock type	2	2.30	0.107
Root C/N	1	6.58	0.012
Bedrock type	2	10.42	<0.001
Root C/N:Bedrock type	2	1.89	0.157
Root C/P	1	0.69	0.411
Bedrock type	2	10.93	<0.001

Variables	*Df*	*F* value	*p* value
Root C/P:Bedrock type	2	0.73	0.487
Root N/P	1	0.24	0.624
Bedrock type	2	10.52	<0.001
Root N/P:Bedrock type	2	0.31	0.731
MBC	1	117.76	<0.001
Bedrock type	2	11.46	<0.001
MBC:Bedrock type	2	1.14	0.325
MBN	1	122.67	<0.001
Bedrock type	2	5.65	0.005
MBN:Bedrock type	2	0.46	0.632
MBP	1	48.82	<0.001
Bedrock type	2	6.02	0.004
MBP:Bedrock type	2	4.17	0.019
MBC/MBN	1	12.75	<0.001
Bedrock type	2	7.23	0.001
MBC/MBN:Bedrock type	2	0.65	0.526
MBC/MBP	1	1.95	0.166
Bedrock type	2	8.97	<0.001
MBC/MBP:Bedrock type	2	1.30	0.277
MBN/MBP	1	0.44	0.511
Bedrock type	2	9.84	<0.001
MBN/MBP:Bedrock type	2	0.65	0.523
CUE	1	5.14	0.026
Bedrock type	2	13.96	<0.001
CUE:Bedrock type	2	7.44	0.001
NUE	1	0.47	0.496
Bedrock type	2	10.36	<0.001
NUE:Bedrock type	2	1.12	0.330
C_{growth}	1	0.88	0.350
Bedrock type	2	9.82	<0.001
C_{growth}:Bedrock type	2	0.45	0.638
q_{Growth}	1	17.38	<0.001
Bedrock type	2	11.69	<0.001
q_{Growth}:Bedrock type	2	6.91	0.002
MBT	1	2.38	0.127
Bedrock type	2	10.11	<0.001
MBT:Bedrock type	2	1.70	0.188
VectorL	1	1.62	0.206
Bedrock type	2	9.05	<0.001
VectorL:Bedrock type	2	0.89	0.414
VectorA	1	2.60	0.110

Variables	*Df*	*F* value	*p* value
Bedrock type	2	8.89	<0.001
VectorA:Bedrock type	2	0.42	0.660
GPB	1	2.54	0.115
Bedrock type	2	10.34	<0.001
GPB:Bedrock type	2	0.55	0.581
GNB	1	3.92	0.051
Bedrock type	2	10.33	<0.001
GNB:Bedrock type	2	0.52	0.595
Fungi	1	2.17	0.145
Bedrock type	2	9.74	<0.001
Fungi:Bedrock type	2	0.25	0.778
Bacteria	1	2.53	0.116
Bedrock type	2	10.18	<0.001
Bacteria:Bedrock type	2	0.57	0.567
Total PLFA	1	1.21	0.274
Bedrock type	2	10.83	<0.001
Total PLFA:Bedrock type	2	2.38	0.098
B/F	1	0.08	0.774
Bedrock type	2	9.82	<0.001
B/F:Bedrock type	2	0.69	0.506
GPB/GNB	1	0.01	0.924
Bedrock type	2	10.15	<0.001
GPB/GNB:Bedrock type	2	1.35	0.264
BG	1	0.25	0.617
Bedrock type	2	13.39	<0.001
BG:Bedrock type	2	5.51	0.006
NAG	1	2.42	0.124
Bedrock type	2	9.37	<0.001
NAG:Bedrock type	2	2.70	0.073
LAP	1	0.40	0.531
Bedrock type	2	12.48	<0.001
LAP:Bedrock type	2	1.89	0.157
AP	1	7.13	0.009
Bedrock type	2	11.16	<0.001
AP:Bedrock type	2	9.62	<0.001
CEL	1	3.27	0.074
Bedrock type	2	8.35	<0.001
CEL:Bedrock type	2	0.59	0.556
BX	1	0.88	0.351
Bedrock type	2	12.06	<0.001
BX:Bedrock type	2	9.68	<0.001

Variables	*Df*	*F* value	*p* value
POX	1	0.77	0.382
Bedrock type	2	11.33	<0.001
POX:Bedrock type	2	2.09	0.130
SOC	1	67.62	<0.001
Bedrock type	2	18.90	<0.001
SOC:Bedrock type	2	1.08	0.345
TN	1	118.87	<0.001
Bedrock type	2	13.06	<0.001
TN:Bedrock type	2	0.46	0.634
TP	1	23.73	<0.001
Bedrock type	2	12.39	<0.001
TP:Bedrock type	2	4.84	0.010
C/N	1	2.93	0.091
Bedrock type	2	8.91	<0.001
C/N:Bedrock type	2	0.05	0.953
C/P	1	1.56	0.215
Bedrock type	2	10.56	<0.001
C/P:Bedrock type	2	0.76	0.469
N/P	1	4.76	0.032
Bedrock type	2	10.26	<0.001
N/P:Bedrock type	2	1.93	0.152
NH_4^+	1	0.02	0.893
Bedrock type	2	10.83	<0.001
NH_4^+:Bedrock type	2	2.00	0.142
NO_3^-	1	8.28	0.005
Bedrock type	2	11.19	<0.001
NO_3^-:Bedrock type	2	4.01	0.022
TDN	1	14.32	<0.001
Bedrock type	2	9.95	<0.001
TDN:Bedrock type	2	0.92	0.401
DOC	1	1.34	0.250
Bedrock type	2	9.90	<0.001
DOC:Bedrock type	2	4.02	0.021
TOP	1	40.43	<0.001
Bedrock type	2	14.30	<0.001
TOP:Bedrock type	2	1.69	0.190
WHC	1	73.73	<0.001
Bedrock type	2	11.67	<0.001
WHC:Bedrock type	2	0.42	0.657
CEC	1	107.43	<0.001
Bedrock type	2	3.25	0.043

Variables	*Df*	*F* value	*p* value
CEC:Bedrock type	2	1.37	0.259
SMC	1	22.02	<0.001
Bedrock type	2	23.46	<0.001
SMC:Bedrock type	2	0.34	0.716
Sand	1	16.59	<0.001
Bedrock type	2	6.95	0.002
Sand:Bedrock type	2	2.86	0.063
Silt	1	6.58	0.012
Bedrock type	2	9.27	<0.001
Silt:Bedrock type	2	3.51	0.034
Clay	1	22.16	<0.001
Bedrock type	2	4.72	0.011
Clay:Bedrock type	2	0.19	0.828
Ca_e	1	79.72	<0.001
Bedrock type	2	2.97	0.057
Ca_e:Bedrock type	2	1.15	0.321
Mg_e	1	10.76	0.001
Bedrock type	2	8.24	<0.001
Mg_e:Bedrock type	2	1.88	0.158
Fe_d	1	1.46	0.231
Bedrock type	2	9.65	<0.001
Fe_d:Bedrock type	2	1.29	0.281
Fe_c	1	2.38	0.127
Bedrock type	2	9.21	<0.001
Fe_c:Bedrock type	2	1.72	0.186
Al_o	1	0.32	0.576
Bedrock type	2	11.15	<0.001
Al_o:Bedrock type	2	4.99	0.009
Fe_o	1	0.20	0.659
Bedrock type	2	9.62	<0.001
Fe_o:Bedrock type	2	0.60	0.549
Al_d	1	1.36	0.247
Bedrock type	2	10.63	<0.001
Al_d:Bedrock type	2	2.58	0.082

For full names of variable abbreviations, see the abbreviations section in the Supplementary Information.

Supplementary Table 14 | Effects of climate, plant, soil, and microbial factors on extracellular polymeric substances (EPS) proteins, evaluated using *p* and *F* values from the general linear model.

Variables	*Df*	*F* value	*p* value
Latitude	1	0.58	0.449
Longitude	1	4.68	0.033
Elevation	1	1.60	0.209
MAT	1	2.45	0.121
MAP	1	5.14	0.026
PET	1	6.20	0.015
ADI	1	4.41	0.038
FRB	1	9.25	0.003
Root C	1	5.48	0.022
Root N	1	19.83	<0.001
Root P	1	1.59	0.212
Root C/N	1	14.65	<0.001
Root C/P	1	2.95	0.090
Root N/P	1	0.19	0.663
MBC	1	47.10	<0.001
MBN	1	41.00	<0.001
MBP	1	10.28	0.002
MBC/MBN	1	8.99	0.004
MBC/MBP	1	0.07	0.794
MBN/MBP	1	0.99	0.323
CUE	1	5.61	0.020
NUE	1	3.93	0.050
C_{growth}	1	0.13	0.724
q_{Growth}	1	20.95	<0.001
MBT	1	5.81	0.018
VectorL	1	4.39	0.039
VectorA	1	1.58	0.212
GPB	1	2.70	0.104
GNB	1	3.10	0.082
Fungi	1	1.12	0.292
Bacteria	1	2.64	0.108
Total PLFA	1	1.89	0.173
B/F	1	0.73	0.395
GPB/GNB	1	0.00	0.999
BG	1	0.44	0.511
NAG	1	0.02	0.877
LAP	1	0.28	0.600
AP	1	1.57	0.213

Variables	*Df*	*F* value	*p* value
CEL	1	0.65	0.422
BX	1	0.11	0.738
POX	1	0.36	0.552
SOC	1	13.01	<0.001
TN	1	50.68	<0.001
TP	1	25.44	<0.001
C/N	1	8.35	0.005
C/P	1	1.51	0.223
N/P	1	0.01	0.936
NH_4^+	1	2.66	0.107
NO_3^-	1	17.49	<0.001
TDN	1	20.31	<0.001
DOC	1	0.97	0.328
TOP	1	34.24	<0.001
WHC	1	31.97	<0.001
CEC	1	10.76	0.001
SMC	1	7.53	0.007
Sand	1	6.86	0.010
Silt	1	6.35	0.013
Clay	1	2.88	0.093
Ca_e	1	10.07	0.002
Mg_e	1	4.10	0.046
Fe_d	1	0.60	0.442
Fe_c	1	0.33	0.567
Al_o	1	0.16	0.690
Fe_o	1	0.39	0.532
Al_d	1	0.65	0.424

For full names of variable abbreviations, see the abbreviations section in the Supplementary Information.

Supplementary Table 15 | Effects of climate, plant, soil, microbial factors, and land use type on extracellular polymeric substances (EPS) proteins, evaluated using *p* and *F* values from the general linear model.

Variables	*Df*	*F* value	*p* value
Latitude	1	0.73	0.395
Land use type	2	8.22	<0.001
Latitude:Land use type	2	5.73	0.005
Longitude	1	5.46	0.022
Land use type	2	7.85	<0.001
Longitude:Land use type	2	1.67	0.194
Elevation	1	1.78	0.186
Land use type	2	6.94	0.002
Elevation:Land use type	2	0.10	0.910
MAT	1	3.20	0.077
Land use type	2	9.10	<0.001
MAT:Land use type	2	6.75	0.002
MAP	1	5.67	0.019
Land use type	2	6.28	0.003
MAP:Land use type	2	0.34	0.714
PET	1	7.72	0.007
Land use type	2	8.70	<0.001
PET:Land use type	2	4.35	0.016
ADI	1	5.15	0.026
Land use type	2	7.68	0.001
ADI:Land use type	2	1.79	0.174
FRB	1	10.22	0.002
Land use type	2	6.60	0.002
FRB:Land use type	2	0.10	0.905
Root C	1	5.88	0.017
Land use type	2	4.48	0.014
Root C:Land use type	2	0.76	0.471
Root N	1	20.30	<0.001
Land use type	2	2.70	0.073
Root N:Land use type	2	0.34	0.714
Root P	1	1.77	0.187
Land use type	2	5.59	0.006
Root P:Land use type	2	0.86	0.429
Root C/N	1	15.60	<0.001
Land use type	2	3.47	0.036
Root C/N:Land use type	2	1.35	0.266
Root C/P	1	3.26	0.075
Land use type	2	5.31	0.007

Variables	*Df*	*F* value	*p* value
Root C/P:Land use type	2	0.67	0.513
Root N/P	1	0.21	0.648
Land use type	2	5.20	0.008
Root N/P:Land use type	2	0.67	0.513
MBC	1	52.10	<0.001
Land use type	2	6.05	0.003
MBC:Land use type	2	0.73	0.486
MBN	1	44.70	<0.001
Land use type	2	5.68	0.005
MBN:Land use type	2	0.39	0.677
MBP	1	12.13	<0.001
Land use type	2	7.92	<0.001
MBP:Land use type	2	2.23	0.114
MBC/MBN	1	9.82	0.002
Land use type	2	5.87	0.004
MBC/MBN:Land use type	2	0.32	0.731
MBC/MBP	1	0.09	0.772
Land use type	2	7.49	0.001
MBC/MBP:Land use type	2	4.92	0.009
MBN/MBP	1	1.18	0.281
Land use type	2	7.08	0.001
MBN/MBP:Land use type	2	3.72	0.028
CUE	1	6.17	0.015
Land use type	2	5.73	0.005
CUE:Land use type	2	0.75	0.474
NUE	1	4.28	0.042
Land use type	2	5.65	0.005
NUE:Land use type	2	0.32	0.729
C_{growth}	1	0.14	0.707
Land use type	2	6.82	0.002
C_{growth}:Land use type	2	0.84	0.436
q_{Growth}	1	22.41	<0.001
Land use type	2	4.33	0.016
q_{Growth}:Land use type	2	0.81	0.450
MBT	1	6.37	0.013
Land use type	2	6.15	0.003
MBT:Land use type	2	0.20	0.823
VectorL	1	4.92	0.029
Land use type	2	6.86	0.002
VectorL:Land use type	2	0.54	0.583
VectorA	1	1.82	0.182

Variables	*Df*	*F* value	*p* value
Land use type	2	7.69	<0.001
VectorA:Land use type	2	0.95	0.389
GPB	1	3.18	0.078
Land use type	2	8.59	<0.001
GPB:Land use type	2	1.44	0.243
GNB	1	3.60	0.061
Land use type	2	8.61	<0.001
GNB:Land use type	2	0.58	0.564
Fungi	1	1.27	0.263
Land use type	2	7.26	0.001
Fungi:Land use type	2	0.62	0.541
Bacteria	1	3.09	0.082
Land use type	2	8.51	<0.001
Bacteria:Land use type	2	1.14	0.325
Total PLFA	1	2.17	0.145
Land use type	2	7.82	<0.001
Total PLFA:Land use type	2	0.90	0.409
B/F	1	0.84	0.364
Land use type	2	7.11	0.001
B/F:Land use type	2	1.21	0.302
GPB/GNB	1	0.00	0.999
Land use type	2	7.15	0.001
GPB/GNB:Land use type	2	2.06	0.134
BG	1	0.49	0.485
Land use type	2	7.01	0.002
BG:Land use type	2	0.64	0.530
NAG	1	0.03	0.870
Land use type	2	6.97	0.002
NAG:Land use type	2	0.33	0.720
LAP	1	0.31	0.577
Land use type	2	7.73	<0.001
LAP:Land use type	2	0.05	0.957
AP	1	1.76	0.188
Land use type	2	6.58	0.002
AP:Land use type	2	0.86	0.427
CEL	1	0.74	0.394
Land use type	2	6.98	0.002
CEL:Land use type	2	0.92	0.404
BX	1	0.13	0.725
Land use type	2	6.74	0.002
BX:Land use type	2	0.23	0.795

Variables	*Df*	*F* value	*p* value
POX	1	0.40	0.531
Land use type	2	6.92	0.002
POX:Land use type	2	0.02	0.982
SOC	1	15.77	<0.001
Land use type	2	9.70	<0.001
SOC:Land use type	2	1.84	0.165
TN	1	57.13	<0.001
Land use type	2	6.48	0.002
TN:Land use type	2	1.25	0.291
TP	1	28.62	<0.001
Land use type	2	7.21	0.001
TP:Land use type	2	0.43	0.655
C/N	1	8.98	0.004
Land use type	2	5.38	0.006
C/N:Land use type	2	0.03	0.975
C/P	1	1.65	0.203
Land use type	2	6.17	0.003
C/P:Land use type	2	0.04	0.961
N/P	1	0.01	0.933
Land use type	2	6.84	0.002
N/P:Land use type	2	0.21	0.814
NH_4^+	1	3.06	0.084
Land use type	2	7.58	<0.001
NH_4^+:Land use type	2	1.28	0.284
NO_3^-	1	19.66	<0.001
Land use type	2	6.63	0.002
NO_3^-:Land use type	2	0.96	0.388
TDN	1	21.84	<0.001
Land use type	2	5.20	0.007
TDN:Land use type	2	0.18	0.835
DOC	1	1.11	0.295
Land use type	2	6.77	0.002
DOC:Land use type	2	1.70	0.188
TOP	1	35.56	<0.001
Land use type	2	3.59	0.032
TOP:Land use type	2	0.14	0.869
WHC	1	36.01	<0.001
Land use type	2	7.32	0.001
WHC:Land use type	2	0.38	0.684
CEC	1	12.40	<0.001
Land use type	2	7.48	0.001

Variables	*Df*	*F* value	*p* value
CEC:Land use type	2	1.41	0.250
SMC	1	8.55	0.004
Land use type	2	7.96	<0.001
SMC:Land use type	2	0.19	0.826
Sand	1	7.61	0.007
Land use type	2	6.88	0.002
Sand:Land use type	2	0.04	0.958
Silt	1	6.99	0.010
Land use type	2	6.36	0.003
Silt:Land use type	2	0.16	0.853
Clay	1	3.24	0.075
Land use type	2	7.32	0.001
Clay:Land use type	2	0.29	0.746
Ca_e	1	11.54	0.001
Land use type	2	7.05	0.001
Ca_e:Land use type	2	1.50	0.229
Mg_e	1	4.64	0.034
Land use type	2	7.65	<0.001
Mg_e:Land use type	2	0.28	0.754
Fe_d	1	0.68	0.414
Land use type	2	7.21	0.002
Fe_d:Land use type	2	0.69	0.505
Fe_c	1	0.37	0.546
Land use type	2	6.81	0.002
Fe_c:Land use type	2	0.05	0.950
Al_o	1	0.20	0.659
Land use type	2	7.60	<0.001
Al_o:Land use type	2	4.24	0.018
Fe_o	1	0.47	0.495
Land use type	2	7.64	<0.001
Fe_o:Land use type	2	1.00	0.055
Al_d	1	0.79	0.377
Land use type	2	8.15	<0.001
Al_d:Land use type	2	3.85	0.025

For full names of variable abbreviations, see the abbreviations section in the Supplementary Information.

Supplementary Table 16 | Effects of climate, plant, soil, microbial factors, and bedrock type on extracellular polymeric substances (EPS) proteins, evaluated using *p* and *F* values from the general linear model.

Variables	*Df*	*F* value	*p* value
Latitude	1	0.64	0.425
Bedrock type	2	1.35	0.266
Latitude:Bedrock type	2	5.57	0.005
Longitude	1	4.72	0.033
Bedrock type	2	1.67	0.195
Longitude:Bedrock type	2	0.71	0.496
Elevation	1	1.56	0.215
Bedrock type	2	0.39	0.678
Elevation:Bedrock type	2	0.63	0.537
MAT	1	2.76	0.100
Bedrock type	2	2.72	0.071
MAT:Bedrock type	2	5.01	0.009
MAP	1	5.03	0.027
Bedrock type	2	1.00	0.373
MAP:Bedrock type	2	0.03	0.968
PET	1	6.91	0.010
Bedrock type	2	5.14	0.008
PET:Bedrock type	2	2.02	0.139
ADI	1	4.84	0.030
Bedrock type	2	5.13	0.008
ADI:Bedrock type	2	1.25	0.291
FRB	1	9.43	0.003
Bedrock type	2	1.99	0.143
FRB:Bedrock type	2	0.87	0.421
Root C	1	5.36	0.023
Bedrock type	2	0.83	0.440
Root C:Bedrock type	2	0.26	0.770
Root N	1	19.22	<0.001
Bedrock type	2	0.34	0.714
Root N:Bedrock type	2	0.33	0.721
Root P	1	1.62	0.208
Bedrock type	2	0.85	0.433
Root P:Bedrock type	2	1.90	0.157
Root C/N	1	14.17	<0.001
Bedrock type	2	0.22	0.800
Root C/N:Bedrock type	2	0.35	0.704
Root C/P	1	2.89	0.093
Bedrock type	2	0.84	0.438

Variables	*Df*	*F* value	*p* value
Root C/P:Bedrock type	2	0.30	0.745
Root N/P	1	0.19	0.667
Bedrock type	2	0.69	0.507
Root N/P:Bedrock type	2	0.57	0.567
MBC	1	48.23	<0.001
Bedrock type	2	2.27	0.109
MBC:Bedrock type	2	0.80	0.452
MBN	1	42.06	<0.001
Bedrock type	2	1.74	0.182
MBN:Bedrock type	2	1.43	0.245
MBP	1	10.13	0.002
Bedrock type	2	0.03	0.974
MBP:Bedrock type	2	1.35	0.266
MBC/MBN	1	8.65	0.004
Bedrock type	2	0.00	0.999
MBC/MBN:Bedrock type	2	0.32	0.728
MBC/MBP	1	0.07	0.797
Bedrock type	2	0.46	0.633
MBC/MBP:Bedrock type	2	0.06	0.938
MBN/MBP	1	0.96	0.330
Bedrock type	2	0.71	0.495
MBN/MBP:Bedrock type	2	0.05	0.956
CUE	1	5.50	0.021
Bedrock type	2	0.97	0.383
CUE:Bedrock type	2	0.18	0.836
NUE	1	3.90	0.052
Bedrock type	2	1.35	0.265
NUE:Bedrock type	2	0.28	0.757
C_{growth}	1	0.12	0.727
Bedrock type	2	0.30	0.744
C_{growth}:Bedrock type	2	0.66	0.519
q_{Growth}	1	20.60	<0.001
Bedrock type	2	0.64	0.529
q_{Growth}:Bedrock type	2	0.61	0.548
MBT	1	5.62	0.020
Bedrock type	2	0.26	0.770
MBT:Bedrock type	2	0.23	0.796
VectorL	1	4.59	0.035
Bedrock type	2	0.04	0.962
VectorL:Bedrock type	2	3.92	0.024
VectorA	1	1.57	0.214

Variables	*Df*	*F* value	*p* value
Bedrock type	2	1.57	0.215
VectorA:Bedrock type	2	0.06	0.944
GPB	1	2.65	0.108
Bedrock type	2	0.44	0.645
GPB:Bedrock type	2	0.64	0.528
GNB	1	3.01	0.086
Bedrock type	2	0.43	0.654
GNB:Bedrock type	2	0.31	0.736
Fungi	1	1.09	0.300
Bedrock type	2	0.28	0.755
Fungi:Bedrock type	2	0.29	0.753
Bacteria	1	2.57	0.113
Bedrock type	2	0.41	0.668
Bacteria:Bedrock type	2	0.37	0.693
Total PLFA	1	1.84	0.179
Bedrock type	2	0.44	0.646
Total PLFA:Bedrock type	2	0.44	0.645
B/F	1	0.75	0.391
Bedrock type	2	0.35	0.704
B/F:Bedrock type	2	2.46	0.092
GPB/GNB	1	0.00	0.999
Bedrock type	2	0.31	0.733
GPB/GNB:Bedrock type	2	0.41	0.665
BG	1	0.46	0.501
Bedrock type	2	0.86	0.426
BG:Bedrock type	2	3.31	0.041
NAG	1	0.02	0.879
Bedrock type	2	0.48	0.621
NAG:Bedrock type	2	0.21	0.812
LAP	1	0.30	0.586
Bedrock type	2	0.85	0.433
LAP:Bedrock type	2	4.61	0.013
AP	1	1.55	0.217
Bedrock type	2	0.29	0.747
AP:Bedrock type	2	1.01	0.369
CEL	1	0.67	0.415
Bedrock type	2	0.10	0.904
CEL:Bedrock type	2	3.36	0.039
BX	1	0.12	0.735
Bedrock type	2	0.27	0.766
BX:Bedrock type	2	2.91	0.060

Variables	*Df*	*F* value	*p* value
POX	1	0.34	0.559
Bedrock type	2	0.40	0.669
POX:Bedrock type	2	0.06	0.938
SOC	1	12.88	<0.001
Bedrock type	2	1.17	0.314
SOC:Bedrock type	2	0.38	0.688
TN	1	49.91	<0.001
Bedrock type	2	0.92	0.404
TN:Bedrock type	2	0.40	0.672
TP	1	25.26	<0.001
Bedrock type	2	0.97	0.383
TP:Bedrock type	2	0.72	0.491
C/N	1	8.32	0.005
Bedrock type	2	0.93	0.398
C/N:Bedrock type	2	0.92	0.401
C/P	1	1.46	0.231
Bedrock type	2	0.23	0.793
C/P:Bedrock type	2	0.24	0.788
N/P	1	0.01	0.937
Bedrock type	2	0.30	0.740
N/P:Bedrock type	2	0.28	0.761
NH_4^+	1	2.63	0.108
Bedrock type	2	0.67	0.515
NH_4^+:Bedrock type	2	0.90	0.409
NO_3^-	1	17.13	<0.001
Bedrock type	2	0.36	0.698
NO_3^-:Bedrock type	2	0.73	0.486
TDN	1	19.87	<0.001
Bedrock type	2	0.22	0.805
TDN:Bedrock type	2	0.81	0.449
DOC	1	0.95	0.333
Bedrock type	2	0.49	0.612
DOC:Bedrock type	2	0.52	0.596
TOP	1	34.53	<0.001
Bedrock type	2	1.68	0.192
TOP:Bedrock type	2	0.70	0.500
WHC	1	32.25	<0.001
Bedrock type	2	2.05	0.136
WHC:Bedrock type	2	0.36	0.698
CEC	1	10.74	0.002
Bedrock type	2	1.62	0.204

Variables	*Df*	*F* value	*p* value
CEC:Bedrock type	2	0.29	0.752
SMC	1	7.69	0.007
Bedrock type	2	2.58	0.082
SMC:Bedrock type	2	0.40	0.674
Sand	1	6.86	0.010
Bedrock type	2	0.12	0.885
Sand:Bedrock type	2	1.87	0.160
Silt	1	6.47	0.013
Bedrock type	2	0.12	0.890
Silt:Bedrock type	2	2.69	0.074
Clay	1	2.76	0.101
Bedrock type	2	0.03	0.969
Clay:Bedrock type	2	0.05	0.956
Ca_e	1	10.10	0.002
Bedrock type	2	1.88	0.159
Ca_e:Bedrock type	2	0.22	0.802
Mg_e	1	4.02	0.048
Bedrock type	2	0.08	0.925
Mg_e:Bedrock type	2	1.14	0.325
Fe_d	1	0.58	0.451
Bedrock type	2	0.23	0.799
Fe_d:Bedrock type	2	0.20	0.823
Fe_c	1	0.32	0.571
Bedrock type	2	0.20	0.816
Fe_c:Bedrock type	2	0.77	0.465
Al_o	1	0.16	0.690
Bedrock type	2	0.30	0.741
Al_o:Bedrock type	2	1.52	0.226
Fe_o	1	0.38	0.539
Bedrock type	2	0.36	0.701
Fe_o:Bedrock type	2	0.25	0.784
Al_d	1	0.64	0.428
Bedrock type	2	0.30	0.743
Al_d:Bedrock type	2	1.04	0.358

For full names of variable abbreviations, see the abbreviations section in the Supplementary Information.

Supplementary Table 17 | Effects of climate, plant, soil, and microbial factors on extracellular polymeric substances carbon to microbial biomass carbon (EPS-C/MBC) ratio, evaluated using *p* and *F* values from the general linear model.

Variables	*Df*	*F* value	*p* value
Latitude	1	1.38	0.243
Longitude	1	3.79	0.055
Elevation	1	4.88	0.030
MAT	1	1.55	0.217
MAP	1	9.09	0.003
PET	1	0.27	0.603
ADI	1	8.17	0.005
FRB	1	4.55	0.036
Root C	1	0.13	0.722
Root N/P	1	0.04	0.847
Root P	1	0.37	0.543
Root C/N	1	0.33	0.566
Root C/P	1	0.06	0.810
Root N/P	1	0.01	0.943
MBC	1	14.55	<0.001
MBN	1	7.26	0.008
MBP	1	5.73	0.019
MBC/MBN	1	3.30	0.072
MBC/MBP	1	0.15	0.702
MBN/MBP	1	1.03	0.312
CUE	1	16.30	<0.001
NUE	1	0.18	0.670
C_{growth}	1	17.60	<0.001
q_{Growth}	1	3.71	0.057
MBT	1	0.96	0.330
VectorL	1	4.15	0.045
VectorA	1	0.25	0.622
GPB	1	0.63	0.429
GNB	1	0.55	0.460
Fungi	1	0.00	0.949
Bacteria	1	0.42	0.521
Total PLFA	1	0.00	0.990
B/F	1	0.13	0.715
GPB/GNB	1	0.31	0.581
BG	1	0.79	0.378
NAG	1	1.94	0.167
LAP	1	0.68	0.413
AP	1	12.48	<0.001

Variables	*Df*	*F* value	*p* value
CEL	1	0.29	0.592
BX	1	4.84	0.030
POX	1	3.29	0.073
SOC	1	12.24	<0.001
TN	1	4.49	0.037
TP	1	0.00	0.997
C/N	1	5.73	0.019
C/P	1	5.50	0.021
N/P	1	1.53	0.220
NH_4^+	1	1.94	0.167
NO_3^-	1	0.11	0.745
TDN	1	1.95	0.166
DOC	1	10.42	0.002
TOP	1	0.80	0.373
WHC	1	3.77	0.055
CEC	1	2.00	0.161
SMC	1	13.49	<0.001
Sand	1	8.12	0.005
Silt	1	5.66	0.020
Clay	1	5.71	0.019
Ca_e	1	2.86	0.094
Mg_e	1	2.04	0.157
Fe_d	1	0.01	0.945
Fe_c	1	0.00	0.985
Al_o	1	1.68	0.199
Fe_o	1	0.05	0.830
Al_d	1	1.74	0.190

For full names of variable abbreviations, see the abbreviations section in the Supplementary Information.

Supplementary Table 18 | Effects of climate, plant, soil, microbial factors, and land use type on extracellular polymeric substances carbon to microbial biomass carbon (EPS-C/MBC) ratio, evaluated using *p* and *F* values from the general linear model.

Variables	*Df*	*F* value	*p* value
Latitude	1	1.44	0.234
Land use type	2	3.30	0.042
Latitude:Land use type	2	0.46	0.631
Longitude	1	4.32	0.041
Land use type	2	5.00	0.009
Longitude:Land use type	2	3.33	0.040
Elevation	1	5.07	0.027
Land use type	2	2.94	0.058
Elevation:Land use type	2	0.80	0.452
MAT	1	1.64	0.204
Land use type	2	3.26	0.043
MAT:Land use type	2	1.42	0.248
MAP	1	9.87	0.002
Land use type	2	3.53	0.034
MAP:Land use type	2	2.31	0.105
PET	1	0.29	0.590
Land use type	2	3.80	0.026
PET:Land use type	2	1.34	0.267
ADI	1	8.36	0.005
Land use type	2	2.37	0.099
ADI:Land use type	2	0.72	0.490
FRB	1	4.65	0.034
Land use type	2	2.31	0.106
FRB:Land use type	2	0.59	0.556
Root C	1	0.14	0.712
Land use type	2	4.23	0.018
Root C:Land use type	2	1.01	0.370
Root N	1	0.04	0.843
Land use type	2	4.18	0.019
Root N:Land use type	2	0.55	0.578
Root P	1	0.41	0.523
Land use type	2	3.95	0.024
Root P:Land use type	2	2.07	0.134
Root C/N	1	0.35	0.556
Land use type	2	3.91	0.024
Root C/N:Land use type	2	0.33	0.719
Root C/P	1	0.07	0.793

Variables	*Df*	*F* value	*p* value
Land use type	2	3.57	0.033
Root C/P:Land use type	2	5.83	0.004
Root N/P	1	0.01	0.941
Land use type	2	2.95	0.059
Root N/P:Land use type	2	2.36	0.102
MBC	1	14.92	<0.001
Land use type	2	2.68	0.074
MBC:Land use type	2	0.47	0.630
MBN	1	7.56	0.007
Land use type	2	3.25	0.043
MBN:Land use type	2	0.60	0.552
MBP	1	6.13	0.015
Land use type	2	3.11	0.050
MBP:Land use type	2	2.00	0.142
MBC/MBN	1	3.41	0.068
Land use type	2	3.38	0.039
MBC/MBN:Land use type	2	0.13	0.879
MBC/MBP	1	0.15	0.695
Land use type	2	3.79	0.027
MBC/MBP:Land use type	2	0.41	0.666
MBN/MBP	1	1.07	0.303
Land use type	2	3.47	0.036
MBN/MBP:Land use type	2	0.26	0.772
CUE	1	16.41	<0.001
Land use type	2	2.13	0.125
CUE:Land use type	2	0.16	0.851
NUE	1	0.19	0.661
Land use type	2	4.11	0.020
NUE:Land use type	2	0.51	0.601
C_{growth}	1	17.53	<0.001
Land use type	2	1.33	0.270
C_{growth}:Land use type	2	0.49	0.613
q_{Growth}	1	3.82	0.054
Land use type	2	3.18	0.046
q_{Growth}:Land use type	2	0.10	0.908
MBT	1	1.01	0.318
Land use type	2	3.66	0.030
MBT:Land use type	2	0.73	0.483
VectorL	1	4.25	0.042
Land use type	2	2.11	0.127
VectorL:Land use type	2	1.04	0.358

Variables	*Df*	*F* value	*p* value
VectorA	1	0.26	0.610
Land use type	2	3.82	0.026
VectorA:Land use type	2	1.29	0.282
GPB	1	0.67	0.414
Land use type	2	3.62	0.031
GPB:Land use type	2	1.48	0.234
GNB	1	0.59	0.446
Land use type	2	3.64	0.030
GNB:Land use type	2	1.16	0.319
Fungi	1	0.00	0.948
Land use type	2	4.09	0.020
Fungi:Land use type	2	0.42	0.656
Bacteria	1	0.44	0.507
Land use type	2	3.74	0.028
Bacteria:Land use type	2	1.35	0.264
Total PLFA	1	0.00	0.990
Land use type	2	4.29	0.017
Total PLFA:Land use type	2	1.24	0.295
B/F	1	0.15	0.708
Land use type	2	3.82	0.026
B/F:Land use type	2	0.48	0.619
GPB/GNB	1	0.32	0.573
Land use type	2	3.73	0.028
GPB/GNB:Land use type	2	0.21	0.809
BG	1	0.84	0.362
Land use type	2	4.86	0.010
BG:Land use type	2	0.17	0.846
NAG	1	2.03	0.158
Land use type	2	3.86	0.025
NAG:Land use type	2	0.15	0.858
LAP	1	0.72	0.400
Land use type	2	4.28	0.017
LAP:Land use type	2	0.35	0.707
AP	1	12.88	<0.001
Land use type	2	2.86	0.063
AP:Land use type	2	0.59	0.559
CEL	1	0.31	0.580
Land use type	2	4.90	0.010
CEL:Land use type	2	0.10	0.903
BX	1	5.16	0.026
Land use type	2	4.49	0.014

Variables	*Df*	*F* value	*p* value
BX:Land use type	2	0.44	0.643
POX	1	3.57	0.062
Land use type	2	3.17	0.047
POX:Land use type	2	2.54	0.085
SOC	1	12.17	<0.001
Land use type	2	1.54	0.220
SOC:Land use type	2	0.18	0.839
TN	1	4.70	0.033
Land use type	2	3.44	0.037
TN:Land use type	2	0.67	0.514
TP	1	0.00	0.997
Land use type	2	4.52	0.014
TP:Land use type	2	0.66	0.521
C/N	1	6.03	0.016
Land use type	2	1.69	0.190
C/N:Land use type	2	2.73	0.071
C/P	1	5.47	0.022
Land use type	2	1.64	0.200
C/P:Land use type	2	0.12	0.888
N/P	1	1.57	0.214
Land use type	2	3.05	0.052
N/P:Land use type	2	0.03	0.975
NH_4^+	1	2.08	0.153
Land use type	2	3.11	0.050
NH_4^+:Land use type	2	2.17	0.120
NO_3^-	1	0.11	0.738
Land use type	2	4.04	0.021
NO_3^-:Land use type	2	0.62	0.540
TDN	1	2.14	0.147
Land use type	2	4.85	0.010
TDN:Land use type	2	1.52	0.226
DOC	1	10.68	0.002
Land use type	2	1.77	0.177
DOC:Land use type	2	1.38	0.258
TOP	1	0.88	0.351
Land use type	2	4.36	0.016
TOP:Land use type	2	2.10	0.129
WHC	1	3.96	0.050
Land use type	2	3.03	0.054
WHC:Land use type	2	1.25	0.292
CEC	1	2.07	0.154

Variables	*Df*	*F* value	*p* value
Land use type	2	3.26	0.043
CEC:Land use type	2	0.28	0.755
SMC	1	13.65	<0.001
Land use type	2	1.82	0.169
SMC:Land use type	2	0.71	0.496
Sand	1	8.17	0.005
Land use type	2	2.08	0.131
Sand:Land use type	2	0.20	0.819
Silt	1	5.76	0.019
Land use type	2	2.75	0.070
Silt:Land use type	2	0.05	0.955
Clay	1	5.79	0.018
Land use type	2	2.26	0.111
Clay:Land use type	2	0.32	0.725
Ca_e	1	2.94	0.090
Land use type	2	2.95	0.058
Ca_e:Land use type	2	0.19	0.825
Mg_e	1	2.09	0.152
Land use type	2	3.01	0.055
Mg_e:Land use type	2	0.12	0.886
Fe_d	1	0.01	0.944
Land use type	2	3.67	0.030
Fe_d:Land use type	2	0.44	0.647
Fe_c	1	0.00	0.984
Land use type	2	3.87	0.025
Fe_c:Land use type	2	0.61	0.544
Al_o	1	1.72	0.193
Land use type	2	2.89	0.061
Al_o:Land use type	2	0.38	0.687
Fe_o	1	0.05	0.825
Land use type	2	4.59	0.013
Fe_o:Land use type	2	0.18	0.838
Al_d	1	1.79	0.185
Land use type	2	2.97	0.057
Al_d:Land use type	2	0.23	0.793

For full names of variable abbreviations, see the abbreviations section in the Supplementary Information.

Supplementary Table 19 | Effects of climate, plant, soil, microbial factors, and bedrock type on extracellular polymeric substances carbon to microbial biomass carbon (EPS-C/MBC) ratio, evaluated using *p* and *F* values from the general linear model.

Variables	*Df*	*F* value	*p* value
Latitude	1	1.56	0.216
Bedrock type	2	7.41	0.001
Latitude:Bedrock type	2	0.27	0.765
Longitude	1	4.51	0.037
Bedrock type	2	9.72	<0.001
Longitude:Bedrock type	2	0.87	0.423
Elevation	1	5.32	0.023
Bedrock type	2	5.85	0.004
Elevation:Bedrock type	2	0.23	0.798
MAT	1	1.73	0.192
Bedrock type	2	6.91	0.002
MAT:Bedrock type	2	0.25	0.778
MAP	1	9.58	0.003
Bedrock type	2	4.37	0.016
MAP:Bedrock type	2	0.03	0.972
PET	1	0.31	0.578
Bedrock type	2	8.24	<0.001
PET:Bedrock type	2	0.06	0.940
ADI	1	8.47	0.005
Bedrock type	2	3.17	0.047
ADI:Bedrock type	2	0.48	0.619
FRB	1	4.94	0.029
Bedrock type	2	5.33	0.007
FRB:Bedrock type	2	0.46	0.632
Root C	1	0.15	0.702
Bedrock type	2	7.20	0.001
Root C:Bedrock type	2	1.60	0.208
Root N	1	0.04	0.836
Bedrock type	2	6.86	0.002
Root N:Bedrock type	2	2.08	0.132
Root P	1	0.40	0.529
Bedrock type	2	4.36	0.016
Root P:Bedrock type	2	0.39	0.679
Root C/N	1	0.40	0.530
Bedrock type	2	6.94	0.002
Root C/N:Bedrock type	2	3.56	0.033
Root C/P	1	0.06	0.802

Variables	*Df*	*F* value	*p* value
Bedrock type	2	4.66	0.012
Root C/P:Bedrock type	2	0.64	0.531
Root N/P	1	0.01	0.942
Bedrock type	2	4.12	0.020
Root N/P:Bedrock type	2	0.11	0.893
MBC	1	15.95	<0.001
Bedrock type	2	5.46	0.006
MBC:Bedrock type	2	0.89	0.416
MBN	1	8.05	0.006
Bedrock type	2	6.60	0.002
MBN:Bedrock type	2	0.33	0.721
MBP	1	6.60	0.012
Bedrock type	2	8.31	<0.001
MBP:Bedrock type	2	0.51	0.604
MBC/MBN	1	3.91	0.051
Bedrock type	2	7.32	0.001
MBC/MBN:Bedrock type	2	2.94	0.058
MBC/MBP	1	0.17	0.682
Bedrock type	2	8.57	<0.001
MBC/MBP:Bedrock type	2	0.06	0.944
MBN/MBP	1	1.21	0.275
Bedrock type	2	9.18	<0.001
MBN/MBP:Bedrock type	2	0.38	0.684
CUE	1	16.63	<0.001
Bedrock type	2	2.71	0.072
CUE:Bedrock type	2	0.19	0.826
NUE	1	0.21	0.647
Bedrock type	2	7.28	0.001
NUE:Bedrock type	2	1.55	0.219
C_{growth}	1	18.40	<0.001
Bedrock type	2	3.34	0.040
C_{growth}:Bedrock type	2	0.72	0.491
q_{Growth}	1	4.05	0.047
Bedrock type	2	5.74	0.005
q_{Growth}:Bedrock type	2	0.30	0.743
MBT	1	1.08	0.302
Bedrock type	2	7.00	0.002
MBT:Bedrock type	2	0.66	0.520
VectorL	1	4.91	0.029
Bedrock type	2	6.81	0.002
VectorL:Bedrock type	2	3.51	0.034

Variables	*Df*	*F* value	*p* value
VectorA	1	0.28	0.598
Bedrock type	2	7.20	0.001
VectorA:Bedrock type	2	1.17	0.315
GPB	1	0.71	0.401
Bedrock type	2	6.94	0.002
GPB:Bedrock type	2	0.84	0.435
GNB	1	0.62	0.435
Bedrock type	2	6.89	0.002
GNB:Bedrock type	2	0.35	0.706
Fungi	1	0.01	0.946
Bedrock type	2	7.23	0.001
Fungi:Bedrock type	2	0.63	0.534
Bacteria	1	0.47	0.496
Bedrock type	2	7.01	0.002
Bacteria:Bedrock type	2	0.66	0.522
Total PLFA	1	0.00	0.989
Bedrock type	2	7.36	0.001
Total PLFA:Bedrock type	2	0.56	0.571
B/F	1	0.15	0.698
Bedrock type	2	7.18	0.001
B/F:Bedrock type	2	0.73	0.484
GPB/GNB	1	0.36	0.552
Bedrock type	2	7.43	0.001
GPB/GNB:Bedrock type	2	1.74	0.182
BG	1	0.91	0.342
Bedrock type	2	8.24	<0.001
BG:Bedrock type	2	1.04	0.357
NAG	1	2.26	0.136
Bedrock type	2	8.75	<0.001
NAG:Bedrock type	2	0.80	0.455
LAP	1	0.77	0.381
Bedrock type	2	8.06	<0.001
LAP:Bedrock type	2	0.49	0.612
AP	1	13.74	<0.001
Bedrock type	2	5.80	0.004
AP:Bedrock type	2	0.76	0.469
CEL	1	0.33	0.569
Bedrock type	2	7.71	<0.001
CEL:Bedrock type	2	0.05	0.947
BX	1	5.43	0.022
Bedrock type	2	7.18	0.001

Variables	*Df*	*F* value	*p* value
BX:Bedrock type	2	0.26	0.769
POX	1	3.75	0.056
Bedrock type	2	6.87	0.002
POX:Bedrock type	2	1.35	0.265
SOC	1	12.97	<0.001
Bedrock type	2	4.38	0.015
SOC:Bedrock type	2	0.30	0.739
TN	1	4.92	0.029
Bedrock type	2	6.36	0.003
TN:Bedrock type	2	0.00	0.999
TP	1	0.00	0.997
Bedrock type	2	7.87	<0.001
TP:Bedrock type	2	2.27	0.109
C/N	1	6.77	0.011
Bedrock type	2	4.94	0.009
C/N:Bedrock type	2	5.29	0.007
C/P	1	6.58	0.012
Bedrock type	2	6.81	0.002
C/P:Bedrock type	2	4.01	0.022
N/P	1	1.85	0.177
Bedrock type	2	8.74	<0.001
N/P:Bedrock type	2	2.77	0.068
NH_4^+	1	2.21	0.141
Bedrock type	2	7.43	0.001
NH_4^+:Bedrock type	2	0.75	0.477
NO_3^-	1	0.12	0.729
Bedrock type	2	7.21	0.001
NO_3^-:Bedrock type	2	0.63	0.535
TDN	1	2.21	0.141
Bedrock type	2	7.17	0.001
TDN:Bedrock type	2	0.72	0.490
DOC	1	11.68	<0.001
Bedrock type	2	6.33	0.003
DOC:Bedrock type	2	1.14	0.324
TOP	1	0.91	0.342
Bedrock type	2	6.91	0.002
TOP:Bedrock type	2	1.42	0.248
WHC	1	4.09	0.046
Bedrock type	2	5.70	0.005
WHC:Bedrock type	2	0.20	0.819
CEC	1	2.23	0.139

Variables	*Df*	*F* value	*p* value
Bedrock type	2	6.43	0.002
CEC:Bedrock type	2	0.85	0.433
SMC	1	13.77	<0.001
Bedrock type	2	2.75	0.070
SMC:Bedrock type	2	0.17	0.844
Sand	1	8.79	0.004
Bedrock type	2	5.31	0.007
Sand:Bedrock type	2	0.41	0.667
Silt	1	6.18	0.015
Bedrock type	2	5.90	0.004
Silt:Bedrock type	2	0.24	0.787
Clay	1	6.42	0.013
Bedrock type	2	5.79	0.004
Clay:Bedrock type	2	1.80	0.172
Ca_e	1	3.20	0.077
Bedrock type	2	6.24	0.003
Ca_e:Bedrock type	2	1.02	0.365
Mg_e	1	2.46	0.121
Bedrock type	2	7.05	0.001
Mg_e:Bedrock type	2	4.18	0.019
Fe_d	1	0.01	0.941
Bedrock type	2	7.18	0.001
Fe_d:Bedrock type	2	2.31	0.106
Fe_c	1	0.00	0.984
Bedrock type	2	7.17	0.001
Fe_c:Bedrock type	2	2.03	0.138
Al_o	1	1.87	0.175
Bedrock type	2	6.27	0.003
Al_o:Bedrock type	2	0.90	0.412
Fe_o	1	0.05	0.820
Bedrock type	2	6.96	0.002
Fe_o:Bedrock type	2	0.60	0.550
Al_d	1	1.96	0.166
Bedrock type	2	6.55	0.002
Al_d:Bedrock type	2	0.93	0.398

For full names of variable abbreviations, see the abbreviations section in the Supplementary Information.

Supplementary References

1. Huang, L. et al. A review of the role of extracellular polymeric substances (EPS) in wastewater treatment systems. *Int. J. Environ. Res. Public Health* **19**, 12191 (2022).

2. Costa, O. Y., Raaijmakers, J. M. & Kuramae, E. E. Microbial extracellular polymeric substances: ecological function and impact on soil aggregation. *Front. Microbiol.* **9**, 1636 (2018).

3. Zhang, M., Wu, Y., Qu, C., Huang, Q. & Cai, P. Microbial extracellular polymeric substances (EPS) in soil: From interfacial behaviour to ecological multifunctionality. *Geo-Bio Interfaces* **1**, e4 (2024).

4. Rillig, M. C. & Mummey, D. L. Mycorrhizas and soil structure. *New Phytol.* **171**, 41–53 (2006).

5. Redmile-Gordon, M., Gregory, A. S., White, R. P. & Watts, C. W. Soil organic carbon, extracellular polymeric substances (EPS), and soil structural stability as affected by previous and current land-use. *Geoderma* **363**, 114143 (2020).

6. Kidinda, L. K. et al. Extracellular polymeric substances are closely related to land cover, microbial communities, and enzyme activity in tropical soils. *Soil Biol. Biochem.* **187**, 109221 (2023).

7. Wang, Z. et al. Human-induced erosion has offset one-third of carbon emissions from land cover change. *Nat. Clim. Change* **7**, 345–349 (2017).

8. Drenovsky, R. E., Steenwerth, K. L., Jackson, L. E. & Scow, K. M. Land use and climatic factors structure regional patterns in soil microbial communities. *Glob. Ecol. Biogeogr.* **19**, 27–39 (2010).

9. Donot, F., Fontana, A., Baccou, J. & Schorr-Galindo, S. Microbial exopolysaccharides: main examples of synthesis, excretion, genetics and extraction. *Carbohydr. Polym.* **87**, 951–962 (2012).

10. Redmile-Gordon, M. A., Brookes, P. C., Evershed, R. P., Goulding, K. W. T. & Hirsch, P. R. Measuring the soil-microbial interface: extraction of extracellular polymeric substances (EPS) from soil biofilms. *Soil Biol. Biochem.* **72**, 163–171 (2014).

11. Zhang, M. et al. Characterising soil extracellular polymeric substances (EPS) by application of spectral-chemometrics and deconstruction of the extraction process. *Chem. Geol.* **618**, 121271 (2023).

12. Chen, Y. et al. Quantifying the trade-off between yield and contamination in soil EPS extraction using cation exchange resin. *J. Soils Sediments* **25**, 2008–2016 (2025).

13. Bérard, A. et al. Exopolysaccharides in the rhizosphere: A comparative study of extraction methods. Application to their quantification in Mediterranean soils. *Soil Biol. Biochem.* **149**, 107961 (2020).

14. Redmile-Gordon, M. & Chen, L. Zinc toxicity stimulates microbial production of extracellular polymers in a copiotrophic acid soil. *Int. Biodeterior. Biodegrad.* **119**, 413–418 (2017).

15. Shi, K. et al. Accumulation of soil microbial extracellular and cellular residues during forest rewilding: Implications for soil carbon stabilization in older plantations. *Soil Biol. Biochem.* **188**, 109250 (2024).

16. Redmile-Gordon, M. A., Evershed, R. P., Hirsch, P. R., White, R. P. & Goulding, K. W. T. Soil organic matter and the extracellular microbial matrix show contrasting responses to C and N availability. *Soil Biol. Biochem.* **88**, 257–267 (2015).

17. Hale, L., Curtis, D., Leon, N., McGiffen Jr, M. & Wang, D. Organic amendments, deficit irrigation, and microbial communities impact extracellular polysaccharide content in agricultural soils. *Soil Biol. Biochem.* **162**, 108428 (2021).

18. Bettermann, A. et al. Importance of microbial communities at the root-soil interface for extracellular polymeric substances and soil aggregation in semiarid grasslands. *Soil Biol. Biochem.* **159**, 108301 (2021).

19. Zethof, J. H. et al. Prokaryotic community composition and extracellular polymeric substances affect soil microaggregation in carbonate containing semiarid grasslands. *Front. Environ. Sci.* **8**, 51 (2020).

20. Baumert, V. L. et al. Root-induced fungal growth triggers macroaggregation in forest subsoils. *Soil Biol. Biochem.* **157**, 108244 (2021).

21. Crouzet, O. et al. Soil photosynthetic microbial communities mediate aggregate stability: influence of cropping systems and herbicide use in an agricultural soil. *Front. Microbiol.* **10**, 1319 (2019).

22. Vuko, M. et al. Shifts in reclamation management strategies shape the role of exopolysaccharide and lipopolysaccharide-producing bacteria during soil formation. *Microb. Biotechnol.* **13**, 584–598 (2020).

23. Liao, J. et al. Unreported role of earthworms as decomposers of soil extracellular polymeric substance. *Appl. Soil Ecol.* **197**, 105325 (2024).

24. Liu, C. et al. Arbuscular mycorrhizal fungi hyphal density rather than diversity stimulates microbial necromass accumulation after long-term *Robinia pseudoacacia* plantations. *Soil Biol. Biochem.* **206**, 109817 (2025).

25. Peng, Y., Zhang, H., Lv, Z., Zhang, J. & Li, G. Microbial inoculation improves soil aggregation by enhancing exopolysaccharides and lipopolysaccharides-related gene abundance in saline soil. *Appl. Soil Ecol.* **214**, 106388 (2025).

26. Bogar, G., Lennon, J., Vander Stel, H. & Evans, S. Microscale assay for the quantification of total polysaccharides to estimate extracellular polymeric substances (EPS) in soil. *J. Microbiol. Methods* **239**, 107324 (2025).

27. Feng, M. et al. Increased microbial extracellular polymeric substances as a key factor in deep soil organic carbon accumulation. *Soil Biol. Biochem.* **212**, 109998 (2026).

28. Li, H. et al. Increased drought intensity stimulates the extracellular polymeric substance accumulation and their contribution to soil organic carbon rather than microbial necromass. *Soil Biol. Biochem.* **213**, 110044 (2026).
