# Supplementary material for: Continental-scale drivers of soil microbial extracellular polymeric substances
Source: Nat Commun. 2026 Mar 2;17:3334. doi: 10.1038/s41467-026-70068-0 (PMC13066494; doi:10.1038/s41467-026-70068-0)
Supplement: Supplementary file 3 — Description of Additional Supplementary Files [file 41467_2026_70068_MOESM3_ESM.pdf]

Description of Additional Supplementary Files

File Name: Supplementary Data 1
Description: Site information, geographic coordinates, and extracellular polymeric substances (EPS) data of the sampled soils.

File Name: Supplementary Data 2
Description: Compilation of published datasets from studies that extracted soil extracellular polymeric substances (EPS) using the cation exchange resin (CER) method.
